# Methylation-mediated silencing of miR-133a-3p promotes breast cancer cell migration and stemness via miR-133a-3p/MAML1/DNMT3A positive feedback loop

**DOI:** 10.1186/s13046-019-1400-z

**Published:** 2019-10-28

**Authors:** Wanyue Shi, Tingting Tang, Xinping Li, Siwei Deng, Ruiyi Li, Yingshan Wang, Yifei Wang, Tiansong Xia, Yanfeng Zhang, Ke Zen, Liang Jin, Yi Pan

**Affiliations:** 1 State Key Laboratory of Natural Medicines, Jiangsu Key Laboratory of Druggability of Biopharmaceuticals, School of Life Science and Technology, China Pharmaceutical University, 24 Tongjiaxiang Avenue, Nanjing, Jiangsu People’s Republic of China; 2 Department of Breast Surgery, Breast Disease Center of Jiangsu Province, First Affiliated Hospital of Nanjing Medical University, 300 Guangzhou Road, Nanjing, Jiangsu People’s Republic of China; 3 Jiangsu Engineering Research Center for microRNA Biology and Biotechnology, State Key Laboratory of Pharmaceutical Biotechnology, School of Life Sciences, Nanjing University, 22 Hankou Road, Nanjing, Jiangsu China

**Keywords:** DNA methylation, miR-133a-3p, Breast cancer, Metastasis, MAML1, DNMT3A

## Abstract

**Background:**

miR-133a-3p has been recently discovered to be down-regulated in various human malignancies, including breast cancer, and reduced miR-133a-3p levels have been significantly associated with breast cancer cell growth and invasion. However, the regulatory mechanisms leading to abnormal expression of miR-133a-3p in breast cancer remain obscure.

**Methods:**

qRT-PCR was applied to detect the expression of miR-133a-3p in breast cancer tissues and cell lines. Bisulfite sequencing was used to detect the degree of methylation of the miR-133a-3p promoter. The effects of miR-133a-3p on breast cancer in vitro were examined by cell proliferation assay, transwell assay, flow cytometry, and western blotting. Bioinformatic analysis, dual-luciferase assay and RIP assay were employed to identify the interaction between miR-133a-3p and MAML1. A xenograft model was used to show the metastasis of breast cancer cells.

**Results:**

We confirmed that miR-133a-3p was silenced by DNA hypermethylation in breast cancer cell lines and tissues, which predicted poor prognosis in breast cancer patients, and reducing miR-133a-3p expression led to a significant increase in the migration, invasion, proliferation, and stemness of breast cancer cells in vitro. Mastermind-like transcriptional coactivator 1 (MAML1) was confirmed to be a target of miR-133a-3p involved in regulating breast cancer metastasis both in vitro and in vivo. Moreover, a series of investigations indicated that MAML1 initiated a positive feedback loop, which could up-regulate DNA methyltransferase 3A (DNMT3A) to promote hypermethylation of the miR-133a-3p promoter.

**Conclusion:**

Taken together, our findings revealed a novel miR-133a-3p/MAML1/DNMT3A positive feedback loop in breast cancer cells, which may become a potential therapeutic target for breast cancer.

**Electronic supplementary material:**

The online version of this article (10.1186/s13046-019-1400-z) contains supplementary material, which is available to authorized users.

## Background

Breast cancer is the most common type of malignant tumor affecting women and it has high incidence and mortality rates worldwide. Although therapeutic interventions have improved in recent years, the clinical outcome of breast cancer patients with distal metastasis and recurrence remains poor [1]. Therefore, an understanding of the molecular mechanisms underlying breast cancer progression, especially metastasis, could provide new therapeutic targets, which may be beneficial for the development of novel therapeutic strategies.

Aberrant expression of microRNAs (miRNAs), which could act as tumor suppressor genes or oncogenes, has been implicated in human carcinogenesis [2–4]. Among them, miR-133a-3p (also named miR-133a) has been reported to down-regulate and display tumor-suppressive function in various human cancers, including bladder cancer, prostate cancer, lung cancer, colon cancer, and breast cancer [5]. Indeed, down-regulation of miR-133a-3p has been found to be associated with disease progression and poor prognosis in breast cancer patients, and the underlying mechanisms have been investigated. For example, miR-133a-3p suppresses tumor cell invasion and migration by targeting Fascin1 (FSCN1) [6] and regulates the cell cycle and proliferation of breast cancer cells by targeting epidermal growth factor receptor (EGFR) [7]. Additionally, miR-133a-3p is involved in doxorubicin-resistance in MCF-7 cells by regulating uncoupling protein 2 (UCP-2, [8]. However, the regulatory mechanisms leading to abnormal expression of miR-133a-3p in breast cancer still need to be studied.

DNA methylation is one of the epigenetic mechanisms controlling gene expression. The methylation of gene promoters is critical for the repression of gene expression in various cell types or development stages [9]. Therefore, abnormal methylation of tumor suppressor genes plays a significant role in tumor development [10, 11], and epigenetic silencing of miRNAs with tumor suppressor features by CpG island hypermethylation has also emerged as a common hallmark of human tumors. For example, miR-200c/141 [12], miR-124a [13], miR-149 [14], miR-193a-3p [15] and miR-375 [16], which are tumor-suppressive miRNAs, are frequently silenced by aberrant DNA hypermethylation in breast cancer genomic DNA.

In the present study, we first validated the down-regulatory expression profile of miR-133a-3p in breast cancer tissues and cells. Then, we demonstrated that DNA methylation mediated miR-133a-3p silencing in breast cancer. Furthermore, in vitro functional assays showed that miR-133a-3p facilitated breast cancer cell migration, invasion, proliferation, and stemness. Mechanistically, we found that mastermind-like transcriptional coactivator 1 (MAML1) is a target of miR-133a-3p. The epigenetic silencing of miR-133a-3p led to the aberrant up-regulation of MAML1, which facilitated the metastasis of breast cancer both in vitro and in vivo. Finally, we revealed that MAML1-up-regulation mediated a positive feedback mechanism by activating the expression of DNA methyltransferases 3A (DNMT3A), which further increased miR-133a-3p promoter methylation. Therefore, the miR-133a-3p/MAML1/DNMT3A positive feedback axis might provide potential targets for breast cancer therapy.

## Materials and methods

### Cell lines and culture

The breast cancer cell lines, MCF-7, MDA-MB-468, and SKBR-3, and the embryonic kidney cell line HEK-293 T were cultured in DMEM medium (Gibco) supplemented with 10% fetal bovine serum (FBS; Gibco). The breast cancer cell line BT-549 was grown in RPMI-1640 medium (Gibco), and the normal human breast cell line MCF-10A was cultured in DMEM/Ham’s F-12 (1:1; Gibco) supplemented with 5% FBS. The breast cancer cell line MDA-MB-231 was cultured in L-15 medium (Gibco) supplemented with 10% FBS. All the cells were purchased from the Institute of Biochemistry and Cell Biology of the Chinese Academy of Sciences (Shanghai, China) and incubated at 37 °C in a humidified incubator containing 5% CO_2_.

### 3D semi-solid spheres culture

Cells were seeded into 24-well Ultra-Low Attachment Microplates (Corning; 3000 cells per well) in serum-free DMEM/F12 (Invitrogen), supplemented with B27 (1:50, Invitrogen), 20 ng/mL EGF (Peprotech), 10 ng/mL bFGF (Invitrogen), 4 μg/mL insulin (Sigma), and 20% methylcellulose (Sigma) respectively. Spheres were collected 7 days after seeding.

### Patients and clinical specimens

All patient samples were collected from the Breast Disease Center of Jiangsu Province, First Affiliated Hospital of Nanjing Medical University (Nanjing, China) with written informed consent. The ethical approval was granted from Committees for Ethical Review in China Pharmaceutical University (Nanjing, China). Pathological diagnosis was made according to the histology of tumor specimens or biopsy and examined by experienced pathologists, and the clinicopathological characteristics are shown in Additional file 3: Table S1. Breast cancer tissues and adjacent normal tissues were stored in liquid nitrogen. The study is compliant with all relevant ethical regulations for human research participants, and all participants provided written informed consent.

### RNA extraction and quantitative RT-PCR

Total RNA was isolated using TRIzol (Invitrogen) and RNeasy kit (QIAGEN) with DNase I digestion according to the manufacturers’ instructions. Reverse transcription reactions were performed using the PrimeScript™ RT reagent Kit (Takara), and diluted cDNA was used for quantitative reverse transcriptase polymerase chain reaction (qRT-PCR) analysis using SYBR Premix Ex Taq II Kit (Takara) with the appropriate primers listed in Additional file 3: Table S2. For miRNA, stem-loop reverse transcription reactions were performed, and the 2^-△Ct^ method was used to calculate the relative abundance of RNA genes compared with glyceraldehyde 3-phosphate dehydrogenase (GAPDH) or U6 expression.

### Bisulfite sequencing

Genomic DNA was extracted from breast cancer cells and subjected to sodium bisulfite modification using EpiTect Bisulfite Kits (QIAGEN) following the manufacturer’s protocols. Sodium bisulfite-treated DNA was PCR amplified using primers (Additional file 3: Table S2) predicted on the MethPrimer website and EpiTaq™HS (Takara). Amplified bisulfate PCR products were subcloned into the TA vector system (Takara) according to the manufacturer’s instructions. For each group, 20 randomly selected clones for specific regions of the gene were sequenced.

### Cell transfection and virus infection

miRNA mimics, inhibitors, small interfering RNAs (siRNAs) (Genepharma) and plasmids transfections were performed using Lipofectamine 2000 (Invitrogen) according to the manufacturer’s protocol. The miR-133a-3p mimics, miR-133a-3p inhibitor, MAML1 siRNA (si-MAML), and their negative controls (NCs) were purchased from Shanghai GenePharma Co., Ltd. (Shanghai, China). The sequences of miR-133a-3p mimics, miR-133a-3p inhibitor, si-MAML1, and their negative controls were list in Additional file 3: Table S3. Lentivirus (Genepharma) encoding miR-133a-3p or MAML1 were imported into MDA-MB-231 cells as previously described [17]. The clones with the stable miR-133a-3p or MAML1 expression were selected by green fluorescence protein (GFP) expression.

### Migration and invasion assays

To evaluate the migration and invasion ability of cells in vitro, wound healing assay, transwell migration assay, and transwell invasion assay were performed based on published methods [17]. For the cell transmembrane migration assay, all the steps were carried out similarly to those in the invasion assay except for the Matrigel (BD Biosciences) coating. After incubation at 37 °C for 24 h or 48 h, the filters were removed. The cells adhering to the lower surface were fixed and stained with 0.1% Crystal Violet (Beyotime). To image the invaded or migrated cells, five randomly selected fields in each well were photographed and counted under an inverted microscope (Olympus) at the magnification of 200. All experiments were performed in triplicate.

### Cell proliferation assay

The capacity of cellular proliferation was measured using the Cell Counting Kit-8 (CCK-8; Bimake) according to the manufacturer’s instructions. Breast cancer cells were harvested after 24 h transfection and re-seeded onto 96-well plates at a density of 3000 cells per well. The optical density was determined with a microplate reader at a wavelength of 450 nm. Each assay was performed in triplicate and independently repeated three times.

### Flow cytometry

Cells were stained using different antibodies according to the manufacturer’s instructions. Labeled cells were detected using a FACS Calibur (BD Immunocytometry Systems). For cell sorting, cocktail PE-conjugated anti-human CD24, APC-conjugated anti-human CD44, and PE/Cy7-conjugated CD326 antibodies were incubated with MCF-7 or MDA-MB-231 cells, followed by sorting with FACS Aria III (BD). Antibodies used are provided in the Additional file 3: Table S4.

### Western blotting

Cells were washed three times with cold phosphate buffered saline (PBS) and total cellular protein was extracted using a modified radioimmunoprecipitation assay (RIPA) lysis buffer on ice. 40 μg of protein proteins were separated with 10% sodium dodecyl sulfate-polyacrylamide gel electrophoresis (SDS-PAGE) and transferred onto 0.22 mm polyvinylidene fluoride membrane (PDVF; Millipore) using a semi-dry transfer cell (Bio-Rad). Antibodies used are listed in Additional file 3: Table S4. The protein bands were visualized using the enhanced chemiluminescence (ECL) detection kit (Tanon). Finally, the proteins were normalized with GAPDH (1:1000; Santa) and visualized by using ECL detection system (Tanon). The blots were analyzed using the ImageJ program.

### Luciferase assay

The putative miR-133a-3p binding sites of the human MAML1 3′-UTR were synthesized and inserted between the SpeI and HindIII sites of the pMIR-REPORT plasmid (MAML1-WT-3′-UTR). We also constructed a pMIR- REPORT plasmid that carried the mutant MAML1 3′-UTR (MAML1-MUT-3′-UTR). For the luciferase reporter assay, HEK-293 T cells were cotransfected with MAML1-WT-3′-UTR or MAML1-MUT-3′-UTR plasmids and mimics NC, miR-133a-3p mimics, inhibitor NC or miR-133a-3p inhibitor by using Lipofectamine 2000 (Invitrogen). The luciferase assay was performed 24 h after transfection using a double-luciferase assay system (Beyotime).

### RNA-binding protein immunoprecipitation (RIP)

For Ago2-based RIP assays, MCF-7 and MDA-MB-231 cells were transfected with pcDNA3.1-MAML1 or pcDNA3.1 empty vector. After 48 h, cells were used to perform RIP experiments using an anti-Ago2 antibody and the Magna RIP™ RNA-Binding Protein Immunoprecipitation Kit (Millipore) according to the manufacturer’s instructions. qRT-PCR was performed to examine the expression levels of MAML1 and miR-133a-3p.

### Xenograft assays in nude mice

Female BALB/c nude mice (5–6 weeks old, 18–20 g) were purchased from the Model Animal Research Center at Nanjing University (Nanjing, China) and kept under specific pathogen-free (SPF) conditions at China Pharmaceutical University. All animal experiments complied with IACUC (Institutional Animal Care and Use Committee) regulations. The subcutaneous xenograft mouse model was used to assess tumor growth. A total of 2 × 10^6^ different infected MCF-7 cells in 0.2 mL PBS were subcutaneously injected into the right armpit region of five-week-old female BALB/c nude mice which were randomly divided into four groups (*n* = 5 per group). Xenograft volumes were evaluated by caliper measurements of two perpendicular diameters and calculated individually by the following: Volume = a × b^2^/2 (a represents length and b represents width). Twenty-four days after injection, the mice were sacrificed and the subcutaneous tumors were isolated and measured. For metastasis experiments, MDA-MB-231 cells were stably transfected with control lentivirus, miR-133a-3p lentivirus, MAML1 lentivirus, or miR-133a-3p plus MAML1 lentivirus. The mice were randomly divided into four groups (5 mice per group), and 1 × 10^6^ cells in 0.1 mL PBS were injected intravenously into the tail. Metastases were then examined by bioluminescence imaging using an IVIS Spectrum Xenogen Imaging System (Xenogen) on day 10, 20 and 30. After scanning, intact lungs and livers were isolated from the mice and photographed. The tissues were excised and embedded in paraffin for histopathological examination. For all animal experiments, the operators and investigators were blinded to the group allocation. All animal experiments were approved by the Ethics Committee of China Pharmaceutical University (Permit Number: 2162326).

### Statistical analysis

All data were shown as the mean and standard error of the mean (mean ± SEM, *n* = 3). Statistical analysis was performed using independent student *t*-test via GraphPad Prism 5.0. *P* < 0.05 was considered statistically significant.

## Results

### miR-133a-3p is down-regulated in breast cancer tissues and cells

**Fig. 1:**
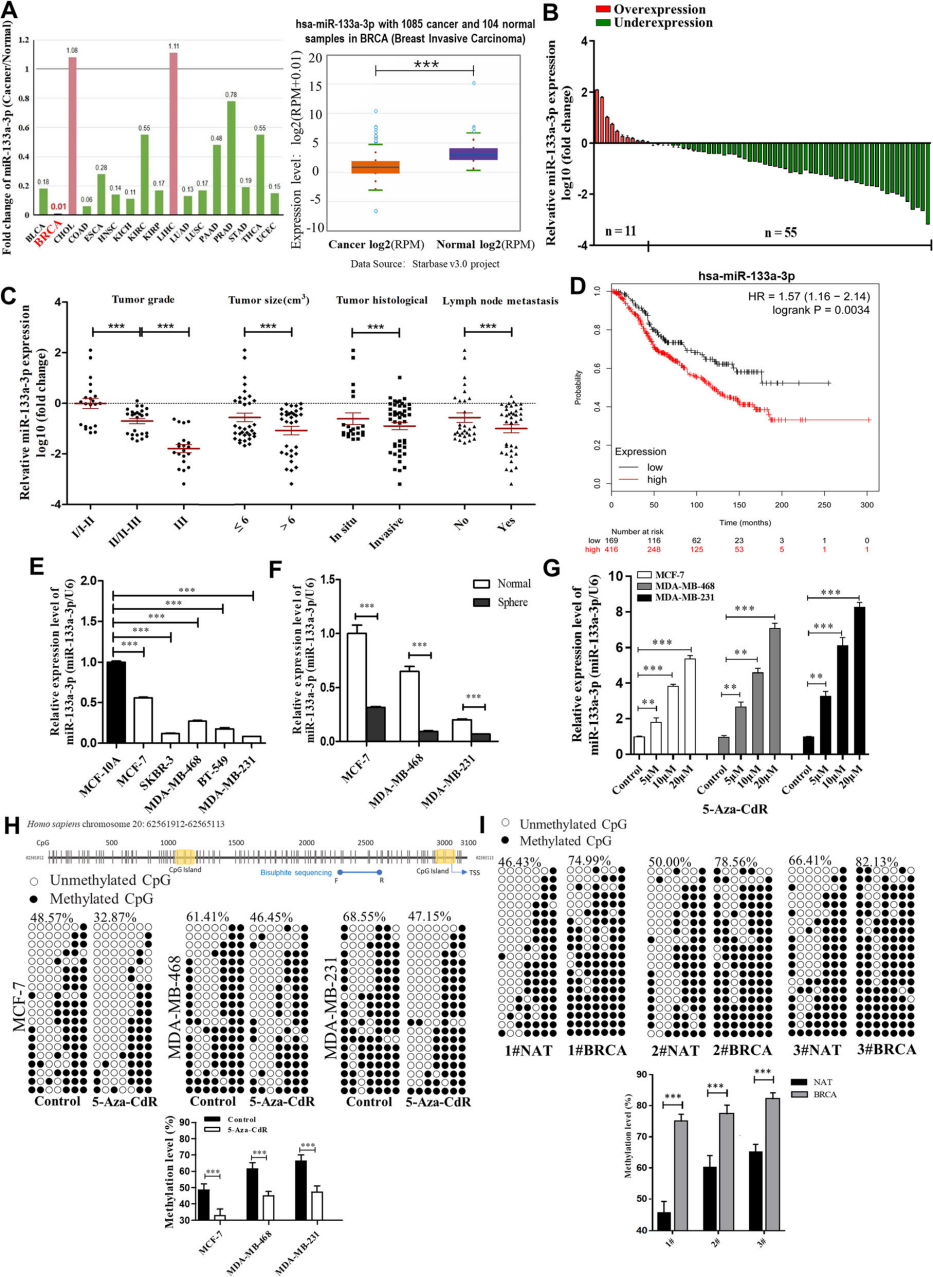
miR-133a-3p is down-regulated in breast cancer cells mediated by DNA methylation. **a** Foldchange of miR-133a-3p in 17 types of cancer tissues compared to their adjacent normal tissues (left) and expression levels of miR-133a-3p in 1085 breast cancer tissues and 104 normal breast tissues (right) in starBase public database from the TCGA project. **b** miR-133a-3p levels were detected in 66 pairs of human breast cancer tissues and corresponding adjacent normal tissues by qRT-PCR. **c** miR-133a-3p levels in breast cancer patients with different tumor grades, sizes and histological, and in patients with different tumor size, with (indicated with ‘yes’) or without (indicated with ‘no’) lymph node metastasis. **d** Overall survival analysis of breast cancer patients with lymph node metastasis based on miR-133a-3p expression (*n* = 585, log-rank test). Data was analyzed using Kaplan Meier Plotter (www.kmplot.com). **e** miR-133a-3p levels in different breast cancer cells and normal breast cells. **f** miR-133a-3p levels in the parental (Normal) or spheroid (Sphere) MCF-7, MDA-MB-468 and MDA-MB-231 cells. **g** miR-133a-3p levels in MCF-7, MDA-MB-468, and MDA-MB-231 cells treated with different concentrations of 5-Aza-CdR for 72 h. **h** A schematic diagram of the CpG island and bisulfite sequencing PCR primers positions in the promoter region of miR-133a-3p (upper) and the methylation levels of miR-133a-3p in MCF-7, MDA-MB-468, and MDA-MB-231 cells treated with 20 μM 5-Aza-CdR for 72 h (lower). Black and white circles represent methylated and unmethylated CpG, respectively. Each row represents an individual sequenced DNA strand. The percentage of methylation in each sequenced region is indicated. **i** The methylation levels of miR-133a-3p in 3 pairs of human breast cancer tissues (indicated by ‘number # BRCA of the patient’) and the corresponding adjacent normal tissues (indicated by ‘number # NAT of the patient’). *P < 0.05; **P < 0.01; ***P < 0.001

By searching the starBase public database, we first determined whether miR-133a-3p had a specific expression profile in breast cancer. We found that miR-133a-3p levels were down-regulated in most (15/17) of the common cancer tissues compared to their normal counterparts, and remarkably lower in 1085 breast cancer tissues compared to 104 normal breast tissues from the TCGA database (Fig. 1a). To validate the down-regulation of miR-133a-3p in breast cancer, we first detected the miR-133a-3p levels in 66 paired breast cancer tissues and adjacent normal tissues by qRT-PCR. As shown in Fig. 1b, miR-133a-3p expression was significantly down-regulated in 83.3% (55 out of 66 pairs) of the breast cancer tissues. We further divided the samples into high (above the median, *n* = 33) and low (below the median, *n* = 33) miR-133a-3p expression groups according to the median value of miR-133a-3p levels and then explored the correlation between miR-133a-3p expression and the clinicopathological characteristics of breast cancer patients. As shown in Additional file 3: Table S1 and Fig. 1c, the miR-133a-3p level was positively associated with tumor grade, tumor size, tumor histological, and lymph node metastasis in this study. Moreover, we evaluated the correlation between miR-133a-3p expression and clinical outcomes from the TCGA database using Kaplan-Meier Plotter (www.kmplot.com). As shown in Fig. 1d, low miR-133a-3p expression predicted poor prognosis in breast cancer patients with lymph node metastasis (*n* = 585, *P* < 0.005).

Furthermore, we detected the expression levels of miR-133a-3p in different breast cancer cell lines (MCF-7, SKBR-3, MDA-MB-468, BT-549, and MDA-MB-231) and a normal breast cell line (MCF-10A). We found that miR-133a-3p levels were not only lower in breast cancer cells than in normal breast cells, but were also decreased more in aggressive breast cancer cells (MDA-MB-231, MDA-MB-468, BT-549, and SKBR-3) than in less aggressive breast cancer cells (MCF-7) (Fig. 1e). Moreover, we cultured sphere breast cancer cells enriched in breast cancer stem cells (BCSCs), which exhibited higher malignancy according to the method previously developed by our laboratory [18], and then measured the expression levels of miR-133a-3p in the sphere and normal breast cancer cells. As shown in Fig. 1f, miR-133a-3p was down-regulated in all three types of sphere breast cancer cells compared with the corresponding normal breast cancer cells. Taken together, these data confirmed that miR-133a-3p is decreased in breast cancer tissues and cells, and may serve as an independent predictor of overall survival in breast cancer and act as a tumor suppressor gene.

### DNA methylation leads to the down-regulation of miR-133a-3p in breast cancer tissues and cells

Next, we explored the mechanisms by which miR-133a-3p expression is down-regulated in breast cancer tissues and cells. Aberrant DNA methylation is a common feature of cancers, which also leads to aberrant expression of some tumor-suppressive miRNAs through the hypermethylation of their promoters [10, 19]. To investigate whether silencing of miR-133a-3p in breast cancer cells was caused by DNA hypermethylation, we treated MCF-7, MDA-MB-468, and MDA-MB-231 cells with different doses of 5-Aza-CdR (5 μM, 10 μM, and 20 μM), a demethylating agent, for 72 h. As shown in Fig. 1g, 5-Aza-CdR induced miR-133a-3p expression in a dose-dependent manner. By searching the MethPrimer database, we identified two CpG islands located at the putative transcription start sites of the miR-133a-3p loci, which suggested that DNA methylation might control the transcriptional activity of miR-133a-3p (Fig. 1h, upper). Then we assessed the extent of CpG methylation within the miR-133a-3p promoter in breast cancer cells and tissues by bisulfite sequencing. As shown, higher degrees of methylation within miR-133a-3p promoter were detected in control cells than in 20 μM 5-Aza-CdR treated MCF-7, MDA-MB-468, and MDA-MB-231 cells, and in MDA-MB-468 and MDA-MB-231 cells than in MCF-7 cells (Fig. 1h, lower). Moreover, we found CpG island hypermethylation of miR-133a-3p in 3 breast cancer tissues and not in the corresponding adjacent normal tissues (Fig. 1i). These results indicated that the expression of miR-133a-3p was down-regulated epigenetically by DNA hypermethylation in breast cancer, which could be restored by treatment with a DNA demethylating agent.

### Silencing of miR-133a-3p expression promotes breast cancer metastasis, proliferation, and stemness

**Fig. 2:**
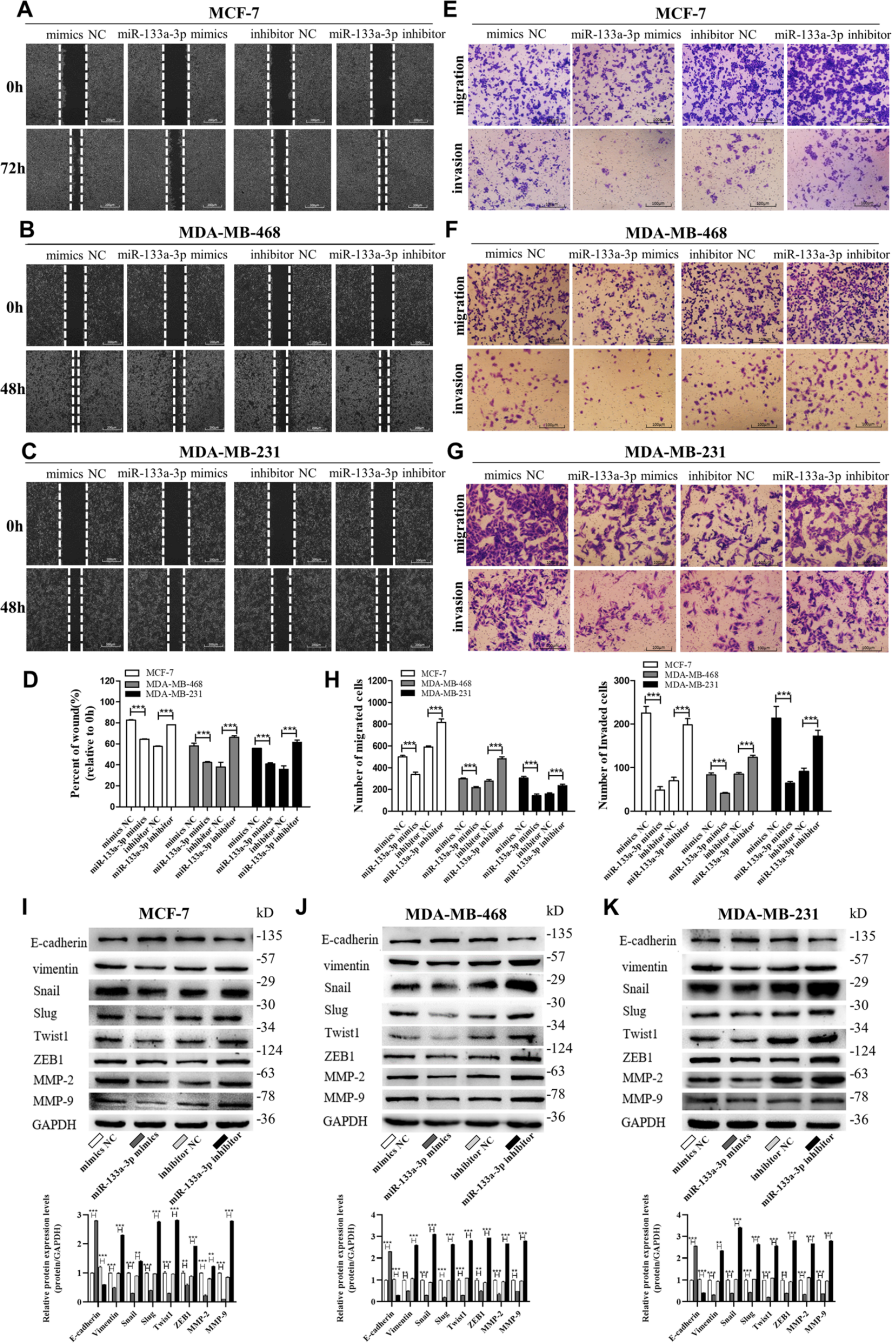
miR-133a-3p promotes breast cancer cells migration and invasion in vitro*.* **a**-**d** Migration of MCF-7 cells (**a**), MDA-MB-468 cells (**b**) and MDA-MB-231 cells (**c**) transfected with mimics NC, miR-133a-3p mimics, inhibitor NC, or miR-133a-3p inhibitor detected by wound healing assay. Analysis of wound closure percentage is from five independent experiments(**d**). Scale bar, 100 μm. **e**-**h** Migration and invasion of MCF-7 cells (**e**), MDA-MB-468 cells (**f**), and MDA-MB-231 cells (**g**) transfected with mimics NC, miR-133a-3p mimics, inhibitor NC, or miR-133a-3p inhibitor detected by transwell migration and invasion assay. Analysis of migrated and invaded cells are from five independent experiments, separately(H). Scale bar, 100 μm. **i**-**k** The protein levels of EMT target genes in MCF-7 cells (**i**), MDA-MB-468 cells (**j**) and MDA-MB-231 cells (**k**) transfected with mimics NC, miR-133a-3p mimics, inhibitor NC or miR-133a-3p inhibitor detected by western blotting. ***P < 0.001

To identify the role of miR-133a-3p in breast cancer progression, we introduced inhibitors or mimics of miR-133a-3p into three breast cancer cell lines (Additional file 1: Figure S1A-C). The wound healing assays (Fig. 2a-d) and transwell assays (Fig. 2e-h) showed that the migration and invasion capacities of MCF-7, MDA-MB-468, and MDA-MB-231 cells were significantly reduced by overexpression of miR-133a-3p but strongly enhanced by depleting miR-133a-3p. We further examined epithelial-mesenchymal transition (EMT)-related proteins in miR-133a-3p overexpressing and knocked-down MCF-7, MDA-MB-468, and MDA-MB-231 cells. As shown in Fig. 2i-j, we found that overexpression of miR-133a-3p inhibited the EMT process in breast cancer cells, whereas knockdown of miR-133a-3p activated EMT, with decreased expression of epithelial markers, and increased expression of mesenchymal markers.

**Fig. 3:**
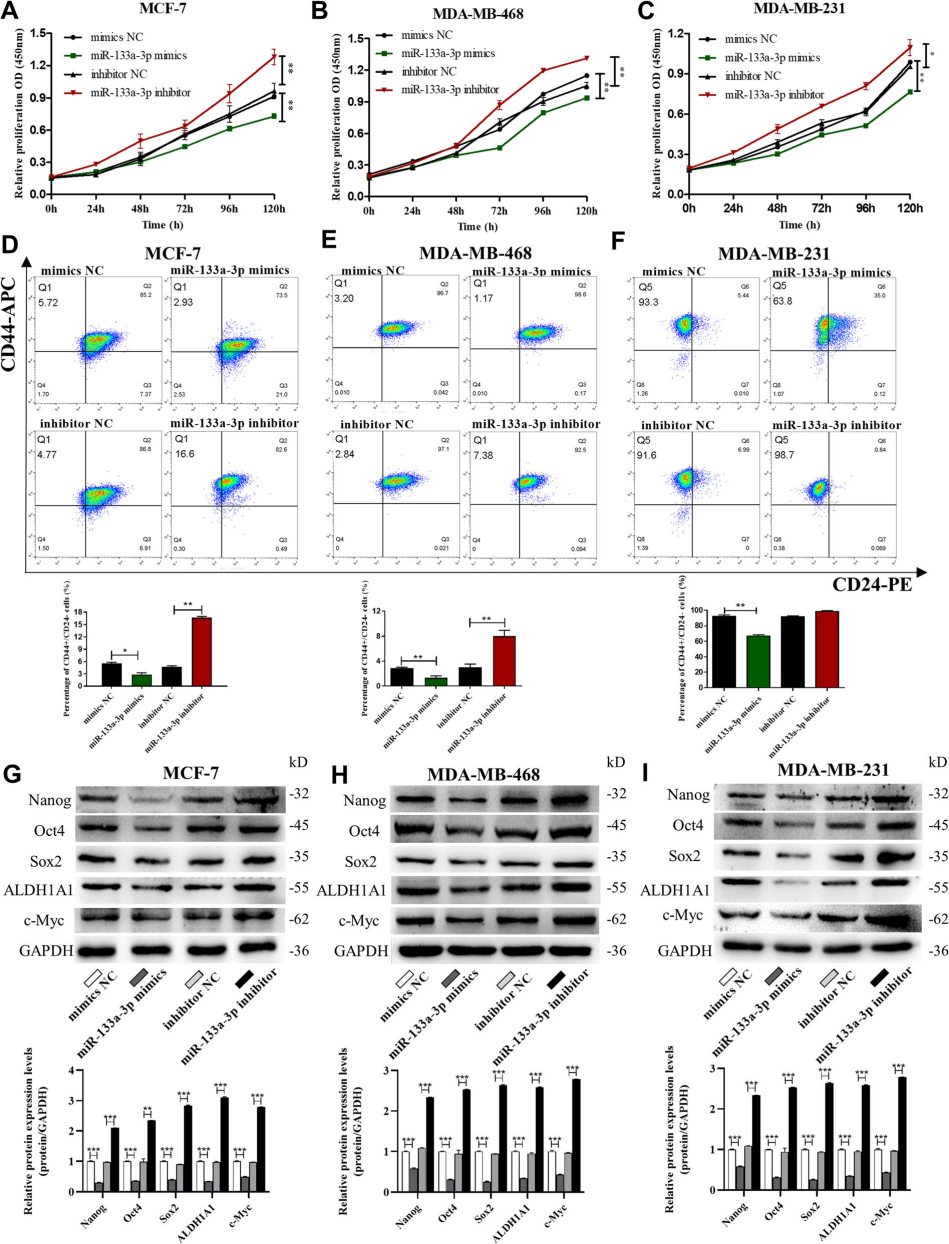
miR-133a-3p promotes breast cancer cells proliferation and stemness in vitro*.* **a**-**c** Proliferation of MCF-7 cells (**a**), MDA-MB-468 cells (**b**) and MDA-MB-231 cells (**c**) transfected with mimics NC, miR-133a-3p mimics, inhibitor NC, or miR-133a-3p inhibitor detected by CCK8 assay. **d**-**f** The percentage of CD44+/CD24- population in MCF-7 cells (**d**), MDA-MB-468 cells (**e**), and MDA-MB-231 cells (**f**) transfected with mimics NC, miR-133a-3p mimics, inhibitor NC or miR-133a-3p inhibitor. **g**-**i** The protein levels of pluripotent transcription factors in MCF-7 cells (**g**), MDA-MB-468 cells (**h**), and MDA-MB-231 cells (**i**) transfected with mimics NC, miR-133a-3p mimics, inhibitor NC, or miR-133a-3p inhibitor by western blotting. *P < 0.05; **P < 0.01; ***P < 0.001

We also studied the effects of miR-133a-3p on breast cancer cell proliferation, stemness, and drug resistance. The CCK-8 assays showed that miR-133a-3p overexpression could significantly inhibit breast cancer cell proliferation, while miR-133a-3p depletion promoted the proliferative abilities of the cells (Fig. 3a-c). Flow cytometric analysis showed that miR-133a-3p overexpression reduced the proportion of BCSCs with CD44+/CD24− phenotype in MCF-7, MDA-MB-468, and MDA-MB-231 cells, while miR-133a-3p knockdown remarkably increased the BCSC proportion (Fig. 3d-f). Notably, miR-133a-3p overexpression and knockdown significantly altered the expression of the pluripotent transcription factors Nanog, Sox2, Oct4, c-Myc, and ALDH1A1 compared with control cells (Fig. G-I). However, overexpression or knockdown of miR-133a-3p in breast cancer cells did not have a significant effect on their drug resistance abilities (data not shown). Our findings suggested that miR-133a-3p could serve as a tumor suppressor gene in breast cancer, and its down-regulation may be required for the migration, invasion, proliferation, and stemness of breast cancer cells.

### Identification of MAML1 as a direct target gene of miR-133a-3p in breast cancer cells

**Fig. 4:**
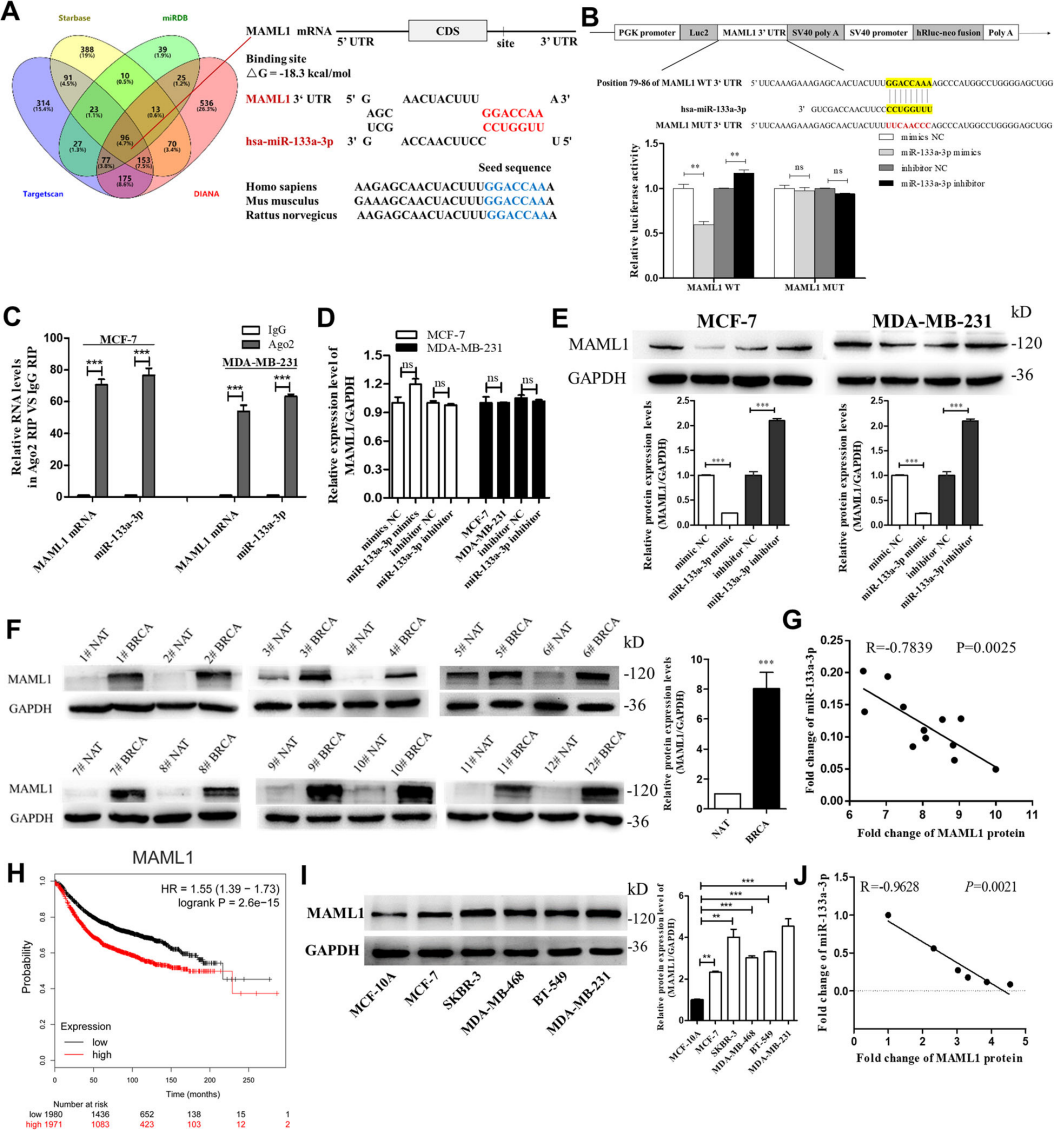
Identification of MAML1 as a direct target gene of miR-133a-3p in breast cancer cells. **a** Left: Venn diagram analysis of four independent databases reveals 96 possible targets of miR-133a-3p. Right: Schematic description of the hypothetical duplexes formed by the interactions between the binding sites in the MAML1 3′-UTR and miR-133a-3p. The predicted free energy value of the hybrid is indicated. The seed recognition sites are denoted, and all nucleotides in these regions are highly conserved across species, including human, mouse, and rat. **b** Luciferase activity in MCF-7 cells co-transfected with a luciferase reporter containing either MAML1-WT or MAML1-MUT (miR-133a-3p-binding sequence mutated), and mimics NC, miR-133a-3p mimics, inhibitor NC or miR-133a-3p inhibitor. Data are presented as the relative ratio of renilla luciferase activity and firefly luciferase activity. **c** Relative enrichment of MAML1 mRNA and miR-133a-3p associated with AGO2 in MCF-7 (left) and MDA-MB-231 (right) cells detected by anti-AGO2 RIP (non-specific IgG as negative control). **d**, **e** MAML1 mRNA (**d**) and protein (**e**) levels in MCF-7 cells and MDA-MB-231 cells transfected with mimics NC, miR-133a-3p mimics, inhibitor NC, or miR-133a-3p inhibitor. **f**, **g** MAML1 protein levels (**f**) and Pearson’s correlation scatter plot of the fold change of miR-133a-3p and MAML1 protein (**g**) in 12 pairs of human breast cancer tissues (BRCA) and corresponding distal non-cancerous tissues (NAT). **h** Overall survival analysis of breast cancer patients based on MAML1 expression (*n* = 3951, log-rank test). Data was analyzed using Kaplan Meier Plotter (www.kmplot.com). **i**, **j** MAML1 protein levels (**i**) and Pearson’s correlation scatter plot of the fold change of miR-133a-3p and MAML1 protein (**j**) in different breast cancer cells and normal breast cells. **P < 0.01; ***P < 0.001

To investigate the underlying molecular mechanisms by which miR-133a-3p promotes breast cancer cell migration, invasion, proliferation, and stemness, we performed Venn diagram analysis to predict miR-133a-3p targets from four independent databases: TargetScan, miRDB, starBase, and DIANA. 96 mRNAs were found in the intersection part (Fig. 4a, left panel). Based on the predetermined conditions (Additional file 3: Table S5) — related to breast cancer, related to cancer migration or invasion and proliferation or stemness [20], has not been reported as a miR-133a-3p target, and correlated with poor survival of breast cancer patients (Kaplan-Meier Plotter) — MAML1,a transcriptional coactivator involved in several signaling pathways mainly activating Notch signaling pathway [21], was therefore selected for further experimental verification. The predicted interaction between miR-133a-3p and its target sites in the MAML1 3′-UTR was illustrated in the right panel of Fig. 4a. There was perfect base-pairing between the seed region and the cognate target. The free energy value of the hybrid was well within the range of genuine miRNA-target pairs (− 18.3 kcal/mol). Subsequently, we confirmed that miR-133a-3p directly targeted the predicted binding sites in the MAML1 3′-UTR by a luciferase reporter assay (Fig. 4b). RIP assays performed with MCF-7 and MDA-MB-231 cell extracts using antibodies against Ago2 demonstrated that MAML1 mRNA and miR-133a-3p were all obviously enriched in Ago2-immunoprecipitation (Ago2-IP) relative to control IgG immunoprecipitation (Fig. 4c). Additionally, we detected that MAML1 mRNA levels had no alteration after either overexpression or knockdown of miR-133a-3p (Fig. 4d), but the MAML1 protein level was significantly decreased in the cells transfected with miR-133a-3p mimics, while remarkably increased by suppression of the endogenous miR-133a-3p (Fig. 4e). The above results suggested that miR-133a-3p down-regulated MAML1 protein level by directly binding to the MAML1 3′-UTR.

Generally, miRNAs have expression patterns that are opposite to those of their targets [22]. Therefore, to investigate whether miR-133a-3p expression was inversely correlated with MAML1 expression in breast cancer cells and clinical specimens, we measured MAML1 protein levels in 12 paired breast cancer tissues and adjacent normal tissues. Notably, we found that MAML1 protein levels were higher in all of the breast cancer tissues than their adjacent normal tissues (Fig. 4f). The inverse correlation between miR-133a-3p levels and MAML1 protein levels (Fig. 4g) was further illustrated using Spearman’s correlation scatter plots, and the correlation between MAML1 mRNA expression and clinical outcomes from the TCGA database was evaluated by using Kaplan Meier Plotter (www.kmplot.com). As shown in Fig. 4h, high MAML1 mRNA expression predicted poor prognosis in breast cancer patients (*n* = 2519, *P* < 0.005). Consistently, MAML1 protein levels were higher in breast cancer cells than in normal breast cells (Fig. 4i), and had a negative correlation with the expression of miR-133a-3p (Fig. 4j). Taken together, these results indicated that MAML1 could be a direct target of miR-133a-3p in breast cancer, and might be corrected with poor outcomes in breast cancer patients.

### miR-133a-3p promotes breast cancer cell migration and invasion via targeting MAML1

**Fig. 5:**
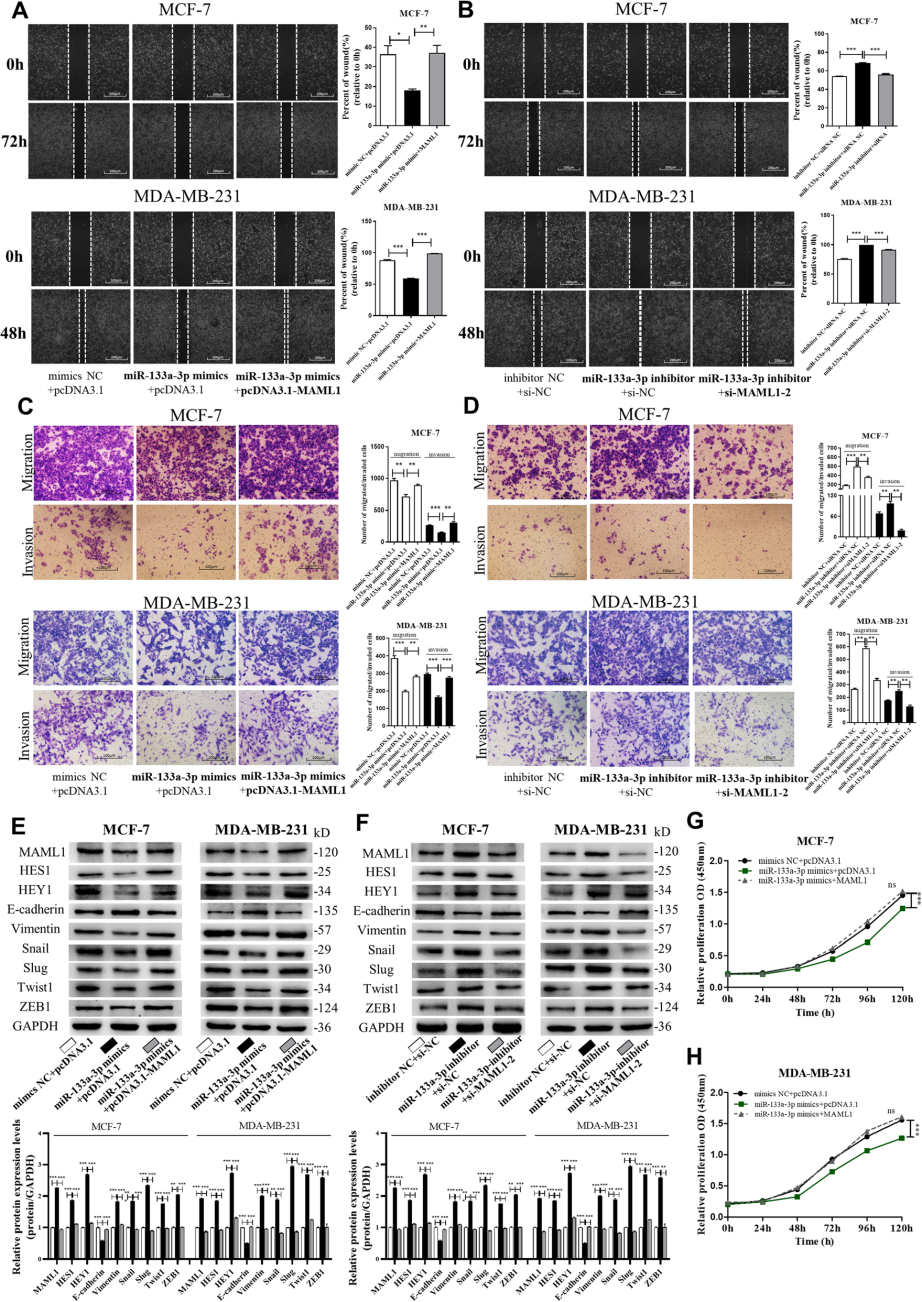
miR-133a-3p promotes breast cancer cells migration and invasion via targeting MAML1. **a**, **b** Migration of MCF-7 cells and MDA-MB-231 cells transfected with mimics NC plus pcDNA3.1, miR-133a-3p mimics plus pcDNA3.1, or miR-133a-3p mimics plus pcDNA3.1-MAML1 (**a**), and inhibitor NC plus si-NC, miR-133a-3p inhibitor plus si-NC, or miR-133a-3p-inhibitor plus si-MAML1–2 (**b**) detected by wound healing assay. Scale bar, 100 μm. **c**, **d** Migration and invasion of MCF-7 cells and MDA-MB-231 cells transfected with mimics NC plus pcDNA3.1, miR-133a-3p mimics plus pcDNA3.1, or miR-133a-3p mimics plus pcDNA3.1-MAML1 (**c**), and inhibitor NC plus si-NC, miR-133a-3p inhibitor plus si-NC, or miR-133a-3p-inhibitor plus si-MAML1–2 (**d**) detected by transwell migration and invasion assay. Scale bar, 100 μm. **e**, **f** The protein levels of MAML1, Notch signaling and EMT target genes in MCF-7 cells (left) and MDA-MB-231 cells (right) transfected with mimics NC plus pcDNA3.1, miR-133a-3p mimics plus pcDNA3.1, or miR-133a-3p mimics plus pcDNA3.1-MAML1 (**e**), and inhibitor NC plus si-NC, miR-133a-3p inhibitor plus si-NC, or miR-133a-3p-inhibitor plus si-MAML1–2 (**f**). **g**, **h** Proliferation of MCF-7 cells (**g**) and MDA-MB-231 cells (**h**) transfected with either the mimics NC plus pcDNA3.1, miR-133a-3p mimics plus pcDNA3.1, or miR-133a-3p mimics plus pcDNA3.1-MAML1 detected by CCK8 assay. *P < 0.05; **P < 0.01; ***P < 0.001

We next focused on studying whether silencing of miR-133a-3p facilitates breast cancer cell migration and invasion by up-regulating MAML1 expression and performed the rescue experiments. We observed that transfecting the MAML1-overexpression plasmid markedly promoted MCF-7 and MDA-MB-231 cell migration and invasion, while transfection of MAML1 siRNAs repressed (Additional file 2: Figure S2A-D). Moreover, compared to cells transfected with miR-133a-3p mimics, the cells transfected with miR-133a-3p mimics and MAML1-overexpression plasmid exhibited significantly higher migration and invasion abilities, while the cells transfected with miR-133a-3p inhibitor and MAML1 siRNA exhibited significantly lower migration and invasion abilities than the cells transfected with miR-133a-3p inhibitor (Fig. 5a-d). These results suggested that MAML1 could attenuate the inhibitory effect of miR-133a-3p on breast cancer cells.

We further investigated whether silencing of miR-133a-3p could activate Notch signaling and the EMT process by up-regulating MAML1. As shown in Fig. 5e and f, overexpression of miR-133a-3p significantly restrained the activation of Notch signaling and EMT, while overexpression of MAML1 abolished the inhibitory effect of miR-133a-3p. In addition, knockdown of MAML1 repressed the promoting effect of miR-133a-3p silencing. Moreover, we observed that MAML1 overexpression slightly promoted the proliferation of breast cancer cells, and attenuated the inhibitory effect of miR-133a-3p on cell proliferation (Fig. 5g and h). Collectively, these data demonstrated that miR-133a-3p silencing could promote the migration, invasion, proliferation, EMT process, and Notch signaling activation in breast cancer cells by targeting MAML1.

### Targeting MAML1 by miR-133a-3p silencing promotes breast tumor cell metastasis in the mouse model

**Fig. 6:**
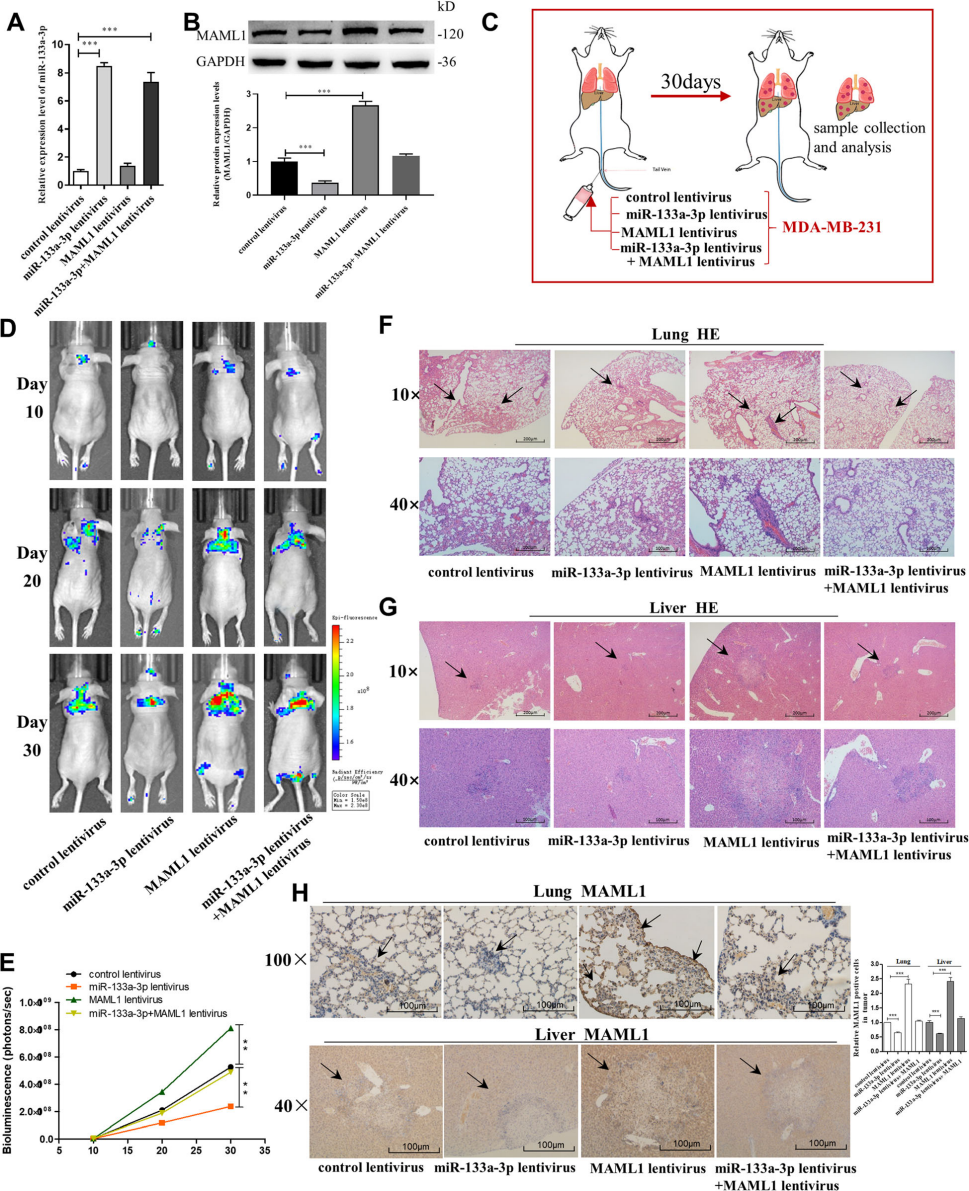
Effects of MAML1-targeted miR-133a-3p on the lung and liver colonization of MDA-MB-231 cells xenografts in mice. **a**, **b** miR-133a-3p levels (**a**) and MAML1 protein levels (**b**) in MDA-MB-231 cells transfected with either the control lentivirus, miR-133a-3p lentivirus, MAML1 lentivirus, or miR-133a-3p lentivirus plus MAML1 lentivirus. **c** Experimental design: immunocompromised mice were injected through tail vein with MDA-MB-231 cells transfected with either the control lentivirus, miR-133a-3p lentivirus, MAML1 lentivirus, or miR-133a-3p lentivirus plus MAML1 lentivirus. **d**, **e** Representative BLI images (**d**) and quantitative analysis of the fluorescence intensities (**e**) of mice of four groups. The BLI was performed on days 10, 20, and 30 after injection. The intensity of BLI is represented by the color. **f**, **g** Representative H&E-stained sections of lung tissues (**f**) and liver tissues (**g**) isolated from the intravenously injected mice. Black arrows indicate metastatic nodules. Scale bar, 200/100 μm. **h** Representative immunohistochemical staining of MAML1 of lung tissues (upper) and liver tissues (lower) respectively. Scale bar, 100 μm. *P < 0.05; **P < 0.01; ***P < 0.001

Next, we investigated the role of miR-133a-3p in mediating breast cancer cell metastasis in the mouse model by targeting MAML1. We first generated four types of modified MDA-MB-231 cell lines: cells infected with control lentivirus, cells stably transfected with miR-133a-3p lentivirus, cells stably transfected with MAML1 lentivirus, and cells stably cotransfected with miR-133a-3p and MAML1 lentiviruses. The qRT-PCR assay and western blot analysis confirmed that the designed four cell lines were successfully established (Fig. 6a and b). Subsequently, we injected these four modified cell lines into female nude mice via the tail vein (Fig. 6c). Metastasis was assessed by bioluminescent imaging (BLI) on day 10, 20, and 30 after implantation. As shown in Fig. 6d and e, the imaging assay indicated that the GFP-labeled migrating cells were mainly distributed in the lungs and livers of the mice. The fluorescent intensities of the lung and liver were significantly weaker in the miR-133a-3p-overexpressing group and stronger in the MAML1-overexpressing group compared to the control. Likewise, MAML1 overexpression attenuated the inhibition of MDA-MB-231 cell metastasis caused by miR-133a-3p-overexpression. Mice were killed after 4 weeks, and the whole lung and liver tissues were harvested, fixed, and sectioned. Then, the tissues were subjected to H&E staining for evaluating tumor metastasis or to immunohistochemical staining for detecting MAML1 expression. The H&E staining results showed significant differences in tumor number and growth in lung and liver tissues (Fig. 6f and g). In mice injected with MAML1 overexpressing MDA-MB-231 cells, larger tumors with clear boundaries (arrows) were found in the lungs and livers, while small masses were scattered in the lungs and livers of the miR-133a-3p overexpression group, and the sizes of tumors in the MAML1 and miR-133a-3p both overexpressed group were similar in comparison with the control group. Moreover, MAML1 labeling revealed that tumors with miR-133a-3p co-overexpression had lower levels of MAML1 than the control group, and tumors with both miR-133a-3p and MAML1 overexpression exhibited higher levels of MAML1 than tumors with miR-133a-3p overexpression (Fig. 6h). These results supported the role of miR-133a-3p in repressing breast cancer cell metastasis in mice through suppressing MAML1 expression.

### Silencing of miR-133a-3p promotes breast tumor growth in vivo by up-regulating MAML1

**Fig. 7:**
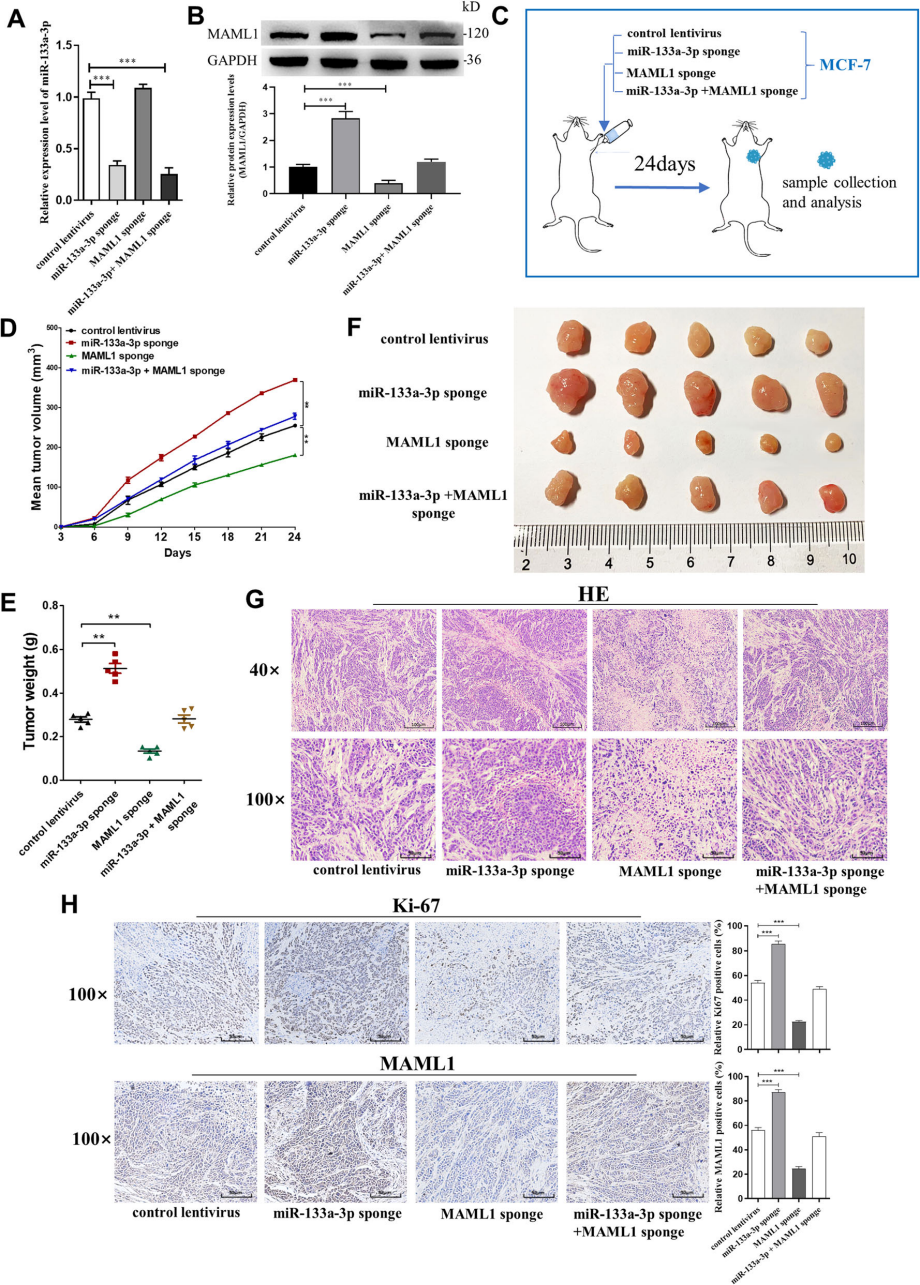
Effects of MAML1-targeted miR-133a-3p on the growth of MCF-7 cell xenografts in mice. **a**, **b** miR-133a-3p levels (**a**) and MAML1 protein levels (**b**) in MCF-7 cells transfected with either the control lentivirus, miR-133a-3p sponge lentivirus, MAML1 sponge lentivirus, or miR-133a-3p sponge lentivirus plus MAML1 sponge lentivirus. **c** Experimental design: the indicated stable MCF-7-expressing cell lines were subcutaneously implanted into nude mice via the armpit. **d**-**f** The tumor growth of mice subcutaneously implanted with the indicated MCF-7 cells. Tumor volume (**d**) and weight (**e**) were measured, and tumor size is pictured (**f**). **g**, **h** Representative H&E-stained sections (**g**) and immunohistochemical staining of Ki-67 and MAML1 of the tumors from subcutaneously implanted mice. Scale bar, 100 μm. *P < 0.05; **P < 0.01; ***P < 0.001

We also evaluated the effects of miR-133a-3p targeting MAML1 on the growth of MCF-7 cell xenografts in mice. MCF-7 cells were transfected with control lentivirus, miR-133a-3p sponge lentivirus, MAML1 sponge lentivirus, or miR-133a-3p sponge lentivirus plus MAML1 sponge lentivirus (Fig. 7a and b). Cells were implanted subcutaneously into the mouse flanks, and tumor growth was evaluated every 3 days after implantation. On day 24, all mice were euthanized, and the tumors were harvested and assessed (Fig. 7c). We observed a significant increase in the size and weight of the tumors in the miR-133a-3p-knockdown xenografts, whereas the size and weight of the tumors in the MAML1-knockdown xenografts was significantly reduced compared with their control groups. Additionally, MAML1-knockdown attenuated the effect of miR-133a-3p-knockdown on tumor growth (Fig. 7d-f). H&E staining showed that the histopathology of tumors formed in the miR-133a-3p-knockdown xenografts exhibited morphologic characteristics of poorly differentiated carcinoma and increased cell mitosis, whereas MAML1-knockdown attenuated these effects (Fig. 7g). The cell proliferation rate, as measured by the percentage of Ki-67+ tumor cells, was increased in tumors from the miR-133a-3p-knockdown xenografts and decreased in tumors from the MAML1-knockdown xenografts. Likewise, MAML1-knockdown attenuated the proliferative effect caused by miR-133a-3p-knockdown (Fig. 7h). Furthermore, miR-133a-3p-knockdown xenografts exhibited higher expression of MAML1 compared with the control, whereas MAML1-knockdown attenuated these effects (Fig. 7h). The above data suggested that silencing of miR-133a-3p may promote tumor growth by up-regulating MAML1.

### MAML1 positive feedback modulates miR-133a-3p promoter methylation through up-regulating DNMT3A

**Fig. 8:**
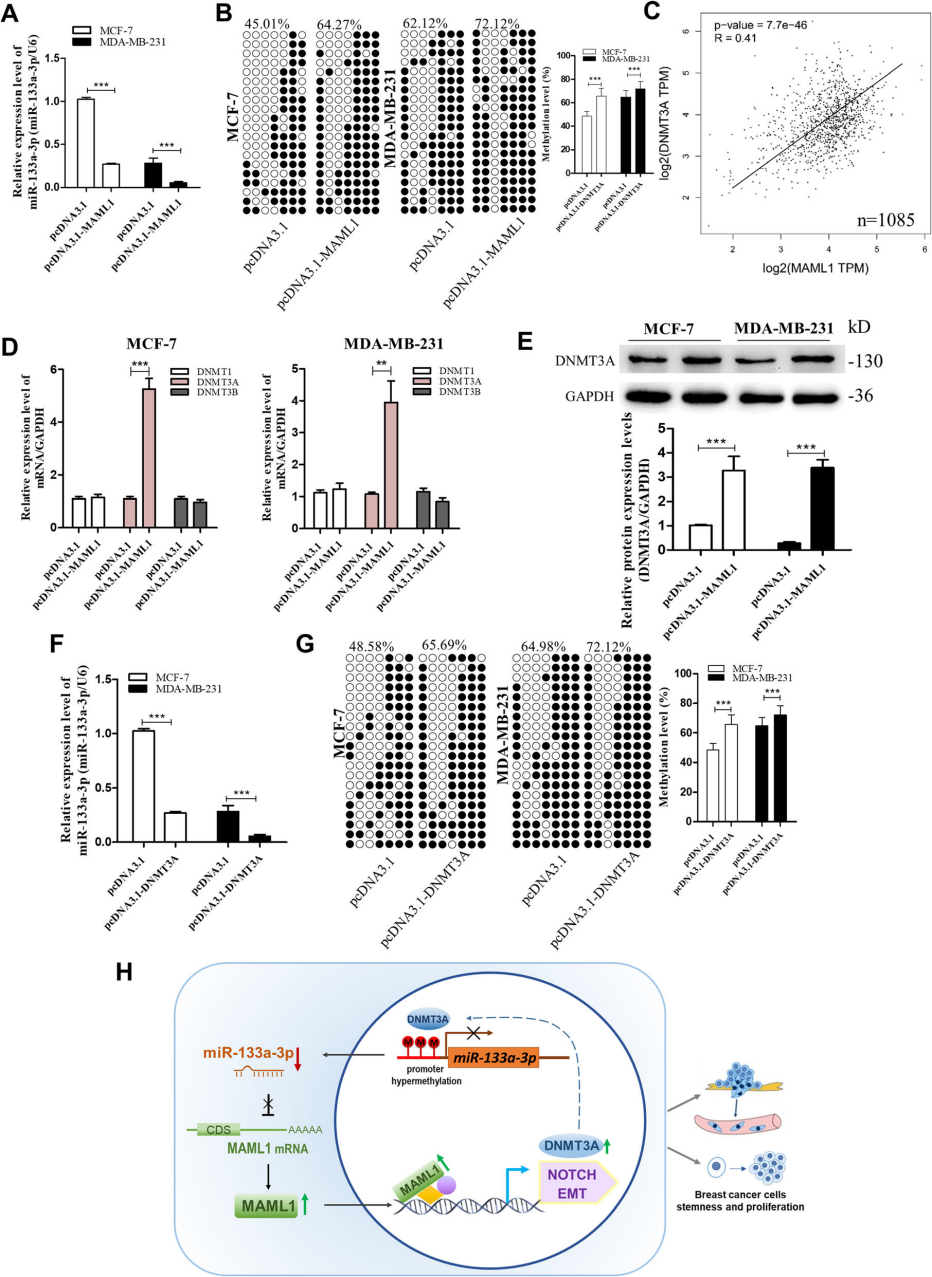
MAML1 feedback regulate methylation of miR-133a-3p through DNMT3A. **a**, **b** mRNA levels (**a**) and the methylation levels (**b**) of miR-133a-3p in MCF-7 cells and MDA-MB-231 cells transfected with either the pcDNA3.1 and pcDNA3.1-MAML1. **c** Pearson’s correlation analysis of the fold change of MAML1 mRNA and DNMT3A mRNA in 1085 human breast cancer tissues by GEPIA database from the TCGA project. **d** The mRNA levels of DNMT1, DNMT3A, and DNMT3B in MCF-7 cells and MDA-MB-231 cells transfected with either the pcDNA3.1 or pcDNA3.1-MAML1. **e** DNMT3A protein levels in MCF-7 cells and MDA-MB-231 cells transfected with either the pcDNA3.1 or pcDNA3.1-MAML1. **f**, **g** mRNA levels (**f**) and the methylation levels (**g**) of miR-133a-3p in MCF-7 cells and MDA-MB-231 cells transfected with either the pcDNA3.1 or pcDNA3.1-DNMT3A. **h** A working model for the role of MAML1-targeted miR-133a-3p in breast cancer metastasis. During breast tumorigenesis, silencing of miR-133a-3p mediated by DNA methylation facilitated proliferation, stemness, migration, and invasion of breast cancer cells through up-regulating the expression of MAML1, which positively provided feedback for the methylation of miR-133a-3p. **P < 0.01; ***P < 0.001

Finally, we determined whether MAML1 could feedback regulate miR-133a-3p promoter methylation since MAML1 functions as a transcriptional coactivator for different transcription factors [23], and thus may activate the transcription of a DNA methyltransferase or demethylase. By detecting miR-133a-3p levels in MCF-7 and MDA-MB-231 cells transfected with pcDNA3.1 and pcDNA3.1-MAML1, we found that miR-133a-3p expression could be down-regulated by MAML1 overexpression (Fig. 8a). Then, we measured the methylation status of the miR-133a-3p promoter in MAML1-overexpressing cells. As shown in Fig. 8b, MAML1 increased the degree of methylation of the miR-133a-3p promoter in both MCF-7 and MDA-MB-231 cells. These data suggested that MAML1 could inhibit miR-133a-3p expression through hypermethylation of the miR-133a-3p promoter.

To explore which type of DNA methyltransferases could be affected by MAML1, we first analyzed the correlation between MAML1 and common DNA methyltransferases (DNMT1, DNMT3A/B) in 1085 breast cancer tissues from the GEPIA public database. Intriguingly, MAML1 mRNA levels only had a significant positive correlation with the expression of DNMT3A (Fig. 8c). By detecting DNMT1, DNMT3A, and DNMT3B mRNA levels (Fig. 8d), as well as DNMT3A protein levels (Fig. 8e) in MAML1-overexpressing breast cancer cells, we confirmed that MAML1 could up-regulate DNMT3A expression. Furthermore, we verified that MAML1 increased the methylation degree of the miR-133a-3p promoter through DNMT3A by overexpressing DNMT3A in MCF-7 and MDA-MB-231 cells and then detecting the expression levels of miR-133a-3p and the methylation status of its promoter region. As shown in Fig. 8f and g, DNMT3A significantly increased the hypermethylation of the miR-133a-3p promoter and therefore suppressed the expression of miR-133a-3p. Taken together, the above results indicated that DNMT3A could be activated by MAML1 and cause the epigenetic silencing of miR-133a-3p.

## Discussion

In this study, we found that the expression of miR-133a-3p was remarkably down-regulated in breast cancer tissues and cells, which was associated with poor prognosis in breast cancer patients. Additionally, we demonstrated that miR-133a-3p was epigenetically suppressed by promoter methylation. In vitro functional assays showed that miR-133a-3p facilitated breast cancer cell migration, invasion, proliferation, and stemness. Mechanistically, we found that MAML1, a transcriptional coactivator of several signaling pathways, including Notch signaling and the EMT process, is a target of miR-133a-3p. miR-133a-3p silencing caused MAML1 up-regulation, leading to the proliferation and metastasis of breast cancer cells both in vitro and in vivo. We also proved a positive feedback loop towards the hypermethylation of miR-133a-3p promoted by MAML1-mediated upregulation of DNMT3A. A working model of our study has been summarized in Fig. 8h.

It is well known that miRNAs often act as oncogenes or tumor suppressors to control many cellular events in tumor development and progression [2, 24]. Recent studies have reported altered expression of miR-133a-3p in several human cancers including esophageal squamous cell carcinoma [25], bladder cancer [26], ileal carcinoid tumor [27] and rhabdomyosarcoma [28]. Ectopic expression of miR-133a-3p significantly inhibits the invasion capacity of various human cancer cell lines [25, 26]. In breast cancer, miR-133a-3p expression was found to be reduced by a microarray-based analysis [29] and significantly downregulated in breast cancer tissues and cell lines [6, 30]. Wu et al. [6] reported that the downregulation of miR-133a was associated with poor relapse-free survival of 90 breast cancer patients and the restoration of miR-133a expression inhibited breast cancer cell growth and invasion in vitro. Similarly, Sui et al. [30] found that miR-133a expression was significantly associated with lymph node and distant metastasis, advanced TNM stage and poor prognosis in breast cancer (*n* = 78). Consistent with these studies, we found the down-regulated behavior of miR-133a-3p in breast cancer by exploring the TCGA database and comparing miR-133a-3p expression levels in 66 breast cancer tissues and five breast cancer cell lines. Strikingly, we found that among 17 common cancer types, miR-133a-3p was downregulated the most in breast cancer, which indicated its important role in breast cancer diagnosis and progression. Moreover, we observed that miR-133a-3p had a lower expression level in breast cancer patients with higher tumor grade, larger tumor size, invasive tumor histology or lymph node metastasis, and low miR-133a-3p expression was associated with shorter overall survival time of breast cancer patients with lymph node metastasis (*n* = 585). Based on our findings and those of others, we suggest that miR-133a-3p might be used as a biomarker for the prediction of breast cancer invasion and metastasis and may play a role as a tumor suppressor gene in regulating cancer development and progression.

Some reports have shown that miR-133a-3p inhibits cancer cell proliferation and migration [6, 30]. However, in vivo experiments have been limited, and few investigations have connected miR-133a-3p with cancer cell stemness or cancer stem cells. Herein, we performed both in vitro and in vivo growth and metastasis analyses. Moreover, we detected that miR-133a-3p was down-regulated in BCSC-enriched spheroid cells, and demonstrated that silencing of miR-133a-3p was involved in the EMT process and stemness inducement of breast cancer cells, which increased the proportion of BCSCs. Extensive studies have demonstrated that BCSCs are the radical cause of malignant proliferation and tumor relapse, and exhibited the ability to metastasize to specific parts of the body, and are believed to be a cause for metastatic lesions [31, 32]. Overall, our data indicated that miR-133a-3p might play an important role in breast cancer cell proliferation and metastasis by inducing more BCSCs.

MAML1, a transcriptional coactivator belonging to the MAML family that is mainly localized in the nucleoplasm, has been reported to be integral to the Notch signaling pathway by regulating the transcriptional activation of Notch target genes, such as HES1 and HEY1. [33]. Notch signaling participates in normal stem cell functions and is also frequently deranged in cancers, which plays a critical role in tumor progression and metastasis by regulating the formation of CSCs and the acquisition of the EMT phenotype [34, 35]. Emerging evidence has implicated MAML1 as an exciting key transcriptional coactivators in other signal transduction pathways, including muscle differentiation and myopathies (MEF2C), tumor suppressor pathway (p53), and colon carcinoma survival (β-catenin). Thus, MAML1 appears to function in transcriptional coactivation in a multitude of cellular processes and human diseases, including cancers [36]. In our study, we proposed that MAML1 was a target of miR-133a-3p and was required for Notch and EMT activation and breast cancer cell proliferation, migration and invasion both in vitro and in vivo. Our data also revealed that MAML1 was up-regulated in breast cancer, and its high expression predicted poor clinical outcome for breast cancer patients.

Down-regulation of miR-133a-3p expression and its tumor suppressor activity in various human cancers are gradually reported [37–39]. However, the molecular mechanisms for miR-133a-3p dysregulation in breast cancer have not been illustrated. Similar to tumor suppressors, dysregulation of tumor suppressor-type miRNAs by epigenetic silencing is often observed in human cancers [40]. We hypothesized that the down-regulated miR-133a-3p was epigenetically silenced by DNA hypermethylation. As expected, hypermethylation of miR-133a-3p could be evidenced using 5-Aza-CdR to mediate the restoration of miR-133a-3p and bisulfite sequencing of CpG islands of the miR-133a-3p promoter. Correspondingly, CpG island hypermethylation of miR-133a-3p was observed in breast cancer tissues and cells. Herein, we were the first to demonstrate that DNA hypermethylation was involved in miR-133a-3p repression in breast cancer. Chen et al. have also reported that DNA hypermethylation leads to silencing of the miR-1-133a cluster expression in colorectal cancer [5]. Combined with our findings, these results suggested that DNA promoter methylation might be a common approach of miR-133a-3p or other miRNA silencing in human cancers. Interestingly, we revealed that miR-133a-3p was epigenetically suppressed by MAML1-induced DNMT3A-mediated promoter methylation, which formed a positive feedback loop that increased the methylation level of the miR-133a-3p promoter, thereby enhancing the pro-proliferation and pro-metastasis effect of miR-133a-3p silencing and MAML1 up-regulation.

## Conclusions

In conclusion, DNA hypermethylation-induced silencing of miR-133a-3p facilitates breast cancer growth and metastasis by up-regulating MAML1, a target of miR-133a-3p. As a transcriptional coactivator, MAML1 promotes DNMT3A expression, which positively regulates miR-133a-3p promoter methylation. We believe that DNA hypermethylation of miR-133a-3p and the miR-133a-3p/MAML1/DNMT3A axis may provide novel therapeutic targets for the treatment of breast cancer, which is also of crucial significance for clinical prevention and diagnosis.

## Additional files

Additional file 1: **Figure S1.** Verification of miR-133a-3p, MAML1 and DNMT3A overexpression or knockdown efficiencies in breast cancer cells. (A-C) miR-133a-3p levels in MCF-7 cells (A), MDA-MB-468 cells (B) and MDA-MB-231 cells (C) transfected with mimics NC, miR-133a-3p mimics, inhibitor NC or miR-133a-3p inhibitor. (D, E) MAML1 mRNA in MCF-7 cells (left) and MDA-MB-231 cells (right) transfected with either the pcDNA3.1 or pcDNA3.1-MAML1(D), either the si-MAML1–1, si-MAML1–2, or si-MAML1–3(E). (F, G) DNMT3A mRNA (F) and protein (G) levels in MCF-7 cells and MDA-MB-231 cells transfected with either the pcDNA3.1 or pcDNA3.1-DNMT3A. **P < 0.01; ***P < 0.001. (TIF 1077 kb)

Additional file 2: **Figure S2.** MAML1 promotes breast cancer cells migration and invasion in vitro. (A, B) Migration of MCF-7 cells (A) and MDA-MB-231 cells (B) transfected with pcDNA3.1, pcDNA3.1-MAML1, si-MAML1–2, or si-MAML1–3 detected by wound healing assay. Scale bar, 100 μm. (C, D) Migration and invasion of MCF-7 cells (C) and MDA-MB-231 cells (D) transfected with pcDNA3.1, pcDNA3.1-MAML1, si-MAML1–2, or si-MAML1–3 detected by transwell migration and invasion assay. Scale bar, 100 μm. (E, F) The proteins levels of the Notch signaling and EMT target gene in MCF-7 cells (left) and MDA-MB-231 cells (right) transfected with pcDNA3.1 or pcDNA3.1-MAML1(E), si-NC, si-MAML1–2 or si-MAML1–3(F) (G, H) Proliferation of MCF-7 cells (G) and MDA-MB-231 cells (H) transfected with pcDNA3.1 or pcDNA3.1-MAML1 detected by CCK-8 assay. *P < 0.05; **P < 0.01; ***P < 0.001. (TIF 4989 kb)

Additional file 3: **Table S1.** miR-133a-3p expression and clinicopathological features in 66 patients with breast cancer. **Table S2.** Sequences of primers used for RT-qPCR, plasmid construction and BSP. **Table S3.** Sequences of mimics, inhibitors and siRNAs. **Table S4.** Antibodies used for western blotting (WB), RNA-binding protein immunoprecipitation (RIP) and flow cytometry (FC). **Table S5.** Screening of 96 predicted targets of miR-133a-3p. (DOCX 43 kb)

## Data Availability

Supporting data includes Supplementary Figures and Supplementary Tables are available.

## Abbreviations

Ago2-IP: Ago2-immunoprecipitation
BCSCs: Breast cancer stem cells
BLI: Bioluminescent imaging
CCK-8: Cell Counting Kit-8
DNMT3A: DNA methyltransferases 3A
ECL: Enhanced chemiluminescence
EGFR: Epidermal growth factor receptor
EMT: Epithelial-mesenchymal transition
FBS: Fetal bovine serum
FSCN1: Fascin1
GAPDH: Glyceraldehyde 3-phosphate dehydrogenase
GFP: Green fluorescence protein
MAML1: Mastermind-like transcriptional co-activator 1
MUT: Mutant
PBS: Phosphate buffered saline
PDVF: Polyvinylidene fluoride membrane
qRT-PCR: Quantitative reverse transcriptase polymerase chain reaction
RIP: RNA-binding protein immunoprecipitation (RIP)
RIPA: Radio-immunoprecipitation
SDS-PAGE: Sulfate-polyacrylamide gel electrophoresis
siRNAs: Small interfering RNAs
SPF: Specific pathogen free
UCP-2: Uncoupling protein 2
WT: Wildtype
