# Supplementary figures and images for: Methylation-mediated silencing of miR-133a-3p promotes breast cancer cell migration and stemness via miR-133a-3p/MAML1/DNMT3A positive feedback loop
Source: J Exp Clin Cancer Res. 2019 Oct 28;38:429. doi: 10.1186/s13046-019-1400-z (PMC6819615; doi:10.1186/s13046-019-1400-z)

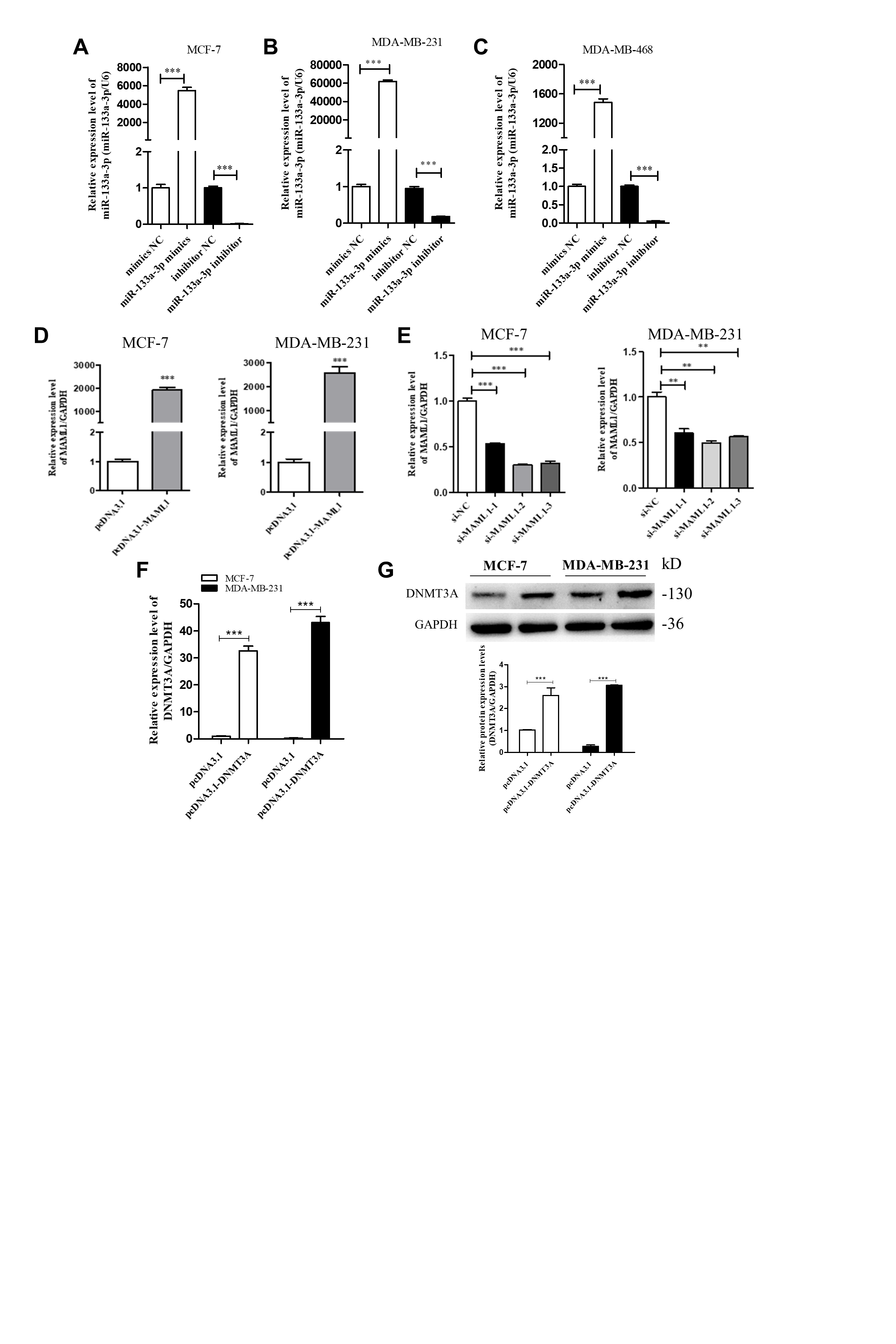

Supplement: Supplementary file 1 — Figure S1. Verification of miR-133a-3p, MAML1 and DNMT3A overexpression or knockdown efficiencies in breast cancer cells. (A-C) miR-133a-3p levels in MCF-7 cells (A), MDA-MB-468 cells (B) and MDA-MB-231 cells (C) transfected with mimics NC, miR-133a-3p mimics, inhibitor NC or miR-133a-3p inhibitor. (D, E) MAML1 mRNA in MCF-7 cells (left) and MDA-MB-231 cells (right) transfected with either the pcDNA3.1 or pcDNA3.1-MAML1(D), either the si-MAML1–1, si-MAML1–2, or si-MAML1–3(E). (F, G) DNMT3A mRNA (F) and protein (G) levels in MCF-7 cells and MDA-MB-231 cells transfected with either the pcDNA3.1 or pcDNA3.1-DNMT3A. **P < 0.01; ***P < 0.001. (TIF 1077 kb) [file 13046_2019_1400_MOESM1_ESM.tif]

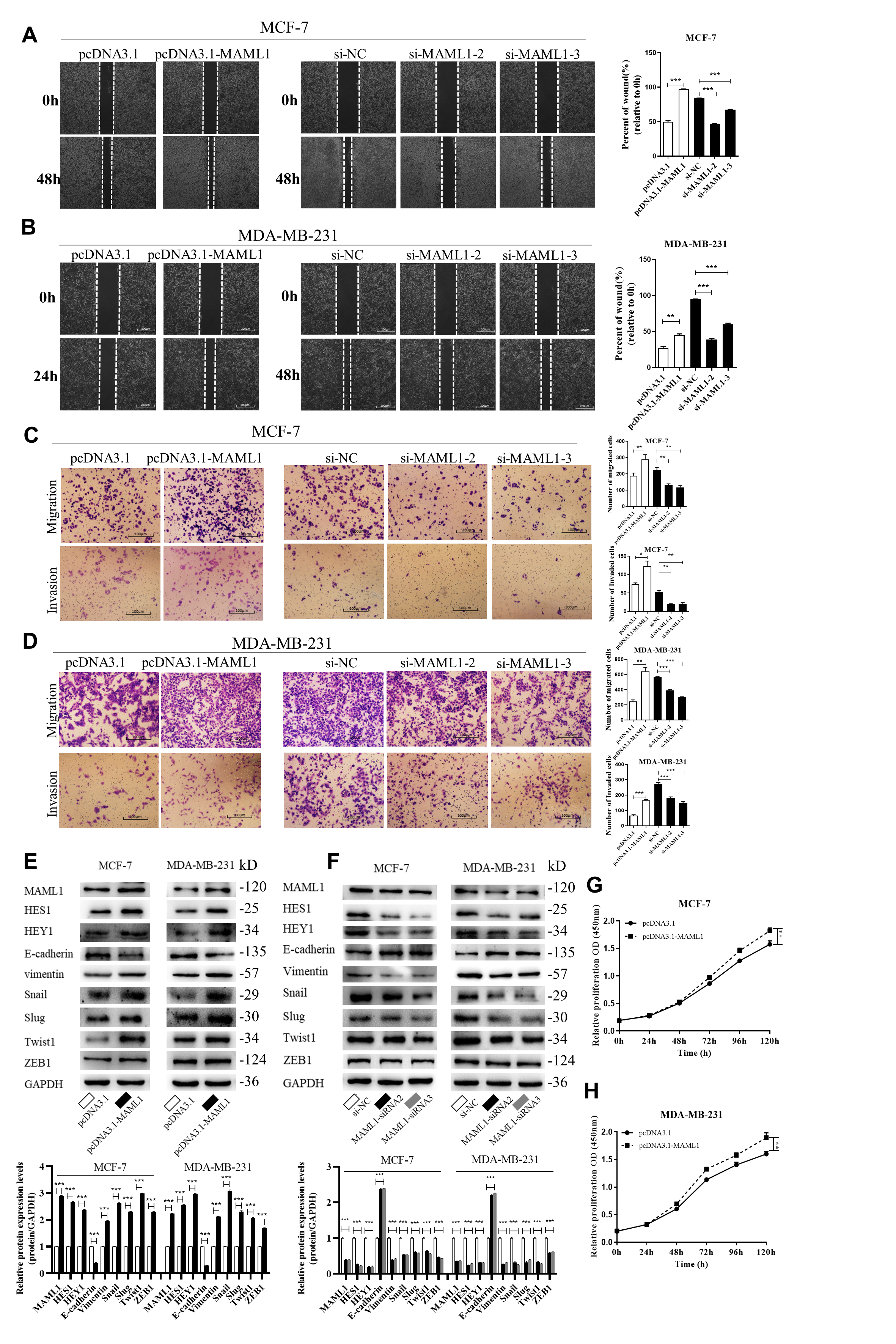

Supplement: Supplementary file 2 — Figure S2. MAML1 promotes breast cancer cells migration and invasion in vitro. (A, B) Migration of MCF-7 cells (A) and MDA-MB-231 cells (B) transfected with pcDNA3.1, pcDNA3.1-MAML1, si-MAML1–2, or si-MAML1–3 detected by wound healing assay. Scale bar, 100 μm. (C, D) Migration and invasion of MCF-7 cells (C) and MDA-MB-231 cells (D) transfected with pcDNA3.1, pcDNA3.1-MAML1, si-MAML1–2, or si-MAML1–3 detected by transwell migration and invasion assay. Scale bar, 100 μm. (E, F) The proteins levels of the Notch signaling and EMT target gene in MCF-7 cells (left) and MDA-MB-231 cells (right) transfected with pcDNA3.1 or pcDNA3.1-MAML1(E), si-NC, si-MAML1–2 or si-MAML1–3(F) (G, H) Proliferation of MCF-7 cells (G) and MDA-MB-231 cells (H) transfected with pcDNA3.1 or pcDNA3.1-MAML1 detected by CCK-8 assay. *P < 0.05; **P < 0.01; ***P < 0.001. (TIF 4989 kb) [file 13046_2019_1400_MOESM2_ESM.tif]
