# Supplementary material for: Methylation-mediated silencing of miR-133a-3p promotes breast cancer cell migration and stemness via miR-133a-3p/MAML1/DNMT3A positive feedback loop
Source: J Exp Clin Cancer Res. 2019 Oct 28;38:429. doi: 10.1186/s13046-019-1400-z (PMC6819615; doi:10.1186/s13046-019-1400-z)
Supplement: Supplementary file 3 — Table S1. miR-133a-3p expression and clinicopathological features in 66 patients with breast cancer. Table S2. Sequences of primers used for RT-qPCR, plasmid construction and BSP. Table S3. Sequences of mimics, inhibitors and siRNAs. Table S4. Antibodies used for western blotting (WB), RNA-binding protein immunoprecipitation (RIP) and flow cytometry (FC). Table S5. Screening of 96 predicted targets of miR-133a-3p. (DOCX 43 kb) [file 13046_2019_1400_MOESM3_ESM.docx]

**Table S1. miR-133a-3p expression and clinicopathological features in 66 patients with breast cancer**

| **Characteristics** | **Expression of miR-133a-3p** | | | ***P*-value*** |  |
| --- | --- | --- | --- | --- | --- |
|  | **low** | | **high** |  |  |
| **Sex** |  | |  |  |  |
| male | 0 | | 0 |  |  |
| female | 33 | | 33 |  |  |
| **Age** |  | |  | 0.616 |  |
| ≤60 | 18 | | 21 |  |  |
| >60 | 15 | | 12 |  |  |
| **Grade** |  | |  | 0.006* |  |
| Ⅰ/I-II, well-differentiated | 7 | | 14 |  |  |
| Ⅱ/II-III, moderately differentiated | 10 | | 15 |  |  |
| Ⅲ, poorly differentiated | 16 | | 4 |  |  |
| **Tumor histological** |  | |  | 0.007* |  |
| Ductal carcinoma in situ | 5 | | 16 |  |  |
| Invasive ductal carcinoma | 28 | | 17 |  |  |
| **T Classification** |  | |  | 0.819 |  |
| T1 | 12 | | 15 |  |  |
| T2 | 10 | | 8 |  |  |
| T3 | 11 | | 10 |  |  |
| **N Classification** |  | |  | 0.146 |  |
| N0 | 7 | | 15 |  |  |
| N1 | 13 | | 7 |  |  |
| N2 | 9 | | 8 |  |  |
| N3 | 4 | | 2 |  |  |
| **ER status** |  | |  | 0.598 |  |
| Negative | 16 | | 19 |  |  |
| Positive | 17 | | 14 |  |  |
| **PR status** |  | |  | 0.798 |  |
| Negative | 22 | | 20 |  |  |
| Positive | 11 | | 13 |  |  |
| **HER2 status** |  | |  | 0.29 |  |
| Negative | 8 | | 13 |  |  |
| Positive | 25 | | 20 |  |  |
| **Tumor size(cm^3^)** |  | |  | 0.042* |  |
| ≤ 6 | 13 | | 21 |  |  |
| > 6 | 20 | | 12 |  |  |
| **Lymph node metastasis** |  | |  | 0.04* |  |
| Negative | 10 | | 19 |  |  |
| Positive | 23 | | 14 |  |  |
| Median expression level was used as a cutoff to divide the 80 patients into miR-133a-3p low group (n = 33) and miR-133a-3p high group (n = 33). | | | | |  |
| Fisher's exact test. **P* < 0.05  **Table S2. Sequences of primers used for RT-qPCR, plasmid construction and BSP** | | | | |  |
| **Primer names** | | **Sequences** | | | |
| **Sequences of primers used for RT-qPCR** | |  | | | |
| hsa-miR-133a-3p forward | | 5'-GCCTTTGGTCCCCTTCAAC-3' | | | |
| hsa-miR-133a-3p reverse | | 5'-TATGCTTGTTCTCGTCTCTGTGTC-3' | | | |
| U6 forward | | 5'-ATTGGAACGATACAGAGAAGATT-3' | | | |
| U6 reverse | | 5'-GGAACGCTTCACGAATTTG-3' | | | |
| MAML1 forward | | 5'-AGACCTCAACCTTAACGAGCA-3' | | | |
| MAML1 reverse | | 5'-TGTGGAGAGCCTAACTGTTCTT-3' | | | |
| GAPDH forward | | 5'-GAGTCAACGGATTTGGTCGT-3' | | | |
| GAPDH reverse | | 5'-TTGATTTTGGAGGGATCTCG-3' | | | |
| DNMT1 forward | | 5'-AGGCGGCTCAAAGATTTGGAA-3' | | | |
| DNMT1 reverse | | 5'-GCAGAAATTCGTGCAAGAGATTC-3' | | | |
| DNMT3A forward | | 5'-CCGATGCTGGGGACAAGAAT-3' | | | |
| DNMT3A reverse | | 5'-CCCGTCATCCACCAAGACAC-3 | | | |
| DNMT3B forward | | 5'-AGGGAAGACTCGATCCTCGTC-3' | | | |
| DNMT3B reverse | | 5'-GTGTGTAGCTTAGCAGACTGG-3' | | | |
| **Sequences of primers used for plasmid construction** | |  | | | |
| pcDNA3.1-MAML1 forward | | 5'-CCCAAGCTTATGGTGCTGCCCACCTGCC-3' | | | |
| pcDNA3.1-MAML1 reverse | | 5'-CGCGGATCCTTACTGAGACCCTAACAGGTCGT-3' | | | |
| pcDNA3.1-DNMA3A forward | | 5'-CCCAAGCTTGCAGTGGGCTCTGGCGGAGGT-3' | | | |
| pcDNA3.1-DNMT3A reverse | | 5'-CGCGGATCCACAGGAAAGCACCAGTACGT-3' | | | |
| pmirGLO-MAML1-WT forward | | 5'-TCGAGTTCAAAGAAAGAGCAACTACTTTGGACCAAAAGCCCATGGC  CTGGGGAGCTGGT-3' | | | |
| pmirGLO-MAML1-WT reverse | | 5'-CTAGACCAGCTCCCCAGGCCATGGGCTTTTGGTCCAAAGTAGTTGCT  CTTTCTTTGAAC-3' | | | |
| pmirGLO-MAML1-MUT forward | | 5'-TCGAGTTCAAAGAAAGAGCAACTACTTTTTCAACCCAGCCCATGGC  CTGGGGAGCTGGT-3' | | | |
| pmirGLO-MAML1-MUT reverse | | 5'-CTAGACCAGCTCCCCAGGCCATGGGCTGGGTTGAAAAAGTAGTTGC  TCTTTCTTTGAAC-3' | | | |
| **Sequences of primers used for BSP** | |  | | | |
| BSP forward | | 5'-TTGTAGGTTTGGGGTATGGG-3' | | | |
| BSP reverse | | 5'-CAACTATAAAATACAACTCAAACCAT-3' | | | |

| **Table S3** **Sequences of mimics, inhibitors and siRNAs** | |
| --- | --- |
| **RNA names** | **Sequences** |
| miR-133a-3p mimics | 5’-UUUGGUCCCCUUCAACCAGCUG-3’ (sense) |
|  | 5’-GCUGGUUGAAGGGGACCAAAUU-3’ (antisense) |
| mimics NC | 5’- UUCUCCGAACGUGUCACGUTT-3’ (sense) |
|  | 5’-ACGUGACACGUUCGGAGAATT-3’ (antisense) |
| miR-133a-3p inhibitor | 5’-CAGCUGGUUGAAGGGGACCAAA-3’ |
| inhibitor NC | 5’-CAGUACUUUUGUGUAGUACAA-3’ |
| MAML1-siRNA-1 | 5‘-CCCUUCCAGCUACAAGCAATT-3’ (Sense) |
|  | 5‘-UUGCUUGUAGCUGGAAGGGTT-3’ (antisense) |
| MAML1-siRNA-2 | 5‘-GCCUUUCCGAUCACUGGUUTT-3’ (Sense) |
|  | 5‘-AACCAGUGAUCGGAAAGGCTT-3’ (antisense) |
| MAML1-siRNA-3 | 5‘-CCUGGACAUGCUUCAGUUUTT-3’ (Sense) |
|  | 5‘-AAACUGAAGCAUGUCCAGGTT-3’ (antisense) |

**Table S4. Antibodies used for western blotting (WB), RNA-binding protein immunoprecipitation (RIP) and flow cytometry (FC).**

| **Protein** | **Applications** | **Antibody** | **Origin** | **dilution** | **Molecular weight** |
| --- | --- | --- | --- | --- | --- |
| GAPDH | WB | D16H11, Cell Signaling Technology | Rabbit | 1:1000 | 36 KD |
| E-cadherin | WB | 3195, Cell Signaling Technology | Rabbit | 1:1000 | 135KD |
| Vimentin | WB | ab92547, Abcam | Rabbit | 1:1000 | 57KD |
| Snail | WB | 3879, Cell Signaling Technology | Rabbit | 1:1000 | 29KD |
| Slug | WB | 9585, Cell Signaling Technology | Rabbit | 1:1000 | 30KD |
| Twist-1 | WB, IP | 46702, Cell Signaling Technology | Rabbit | 1:1000 | 34KD |
| ZEB-1 | WB | 3396, Cell Signaling Technology | Rabbit | 1:1000 | 124KD |
| CD44 | FC | 559942, BD Pharmingen™ | Mouse | 1:200 | 81KD |
| CD24 | FC | 555428, BD Pharmingen™ | Mouse | 1:200 | 9KD |
| Nanog | WB | sc-293121, Santa cruz | Mouse | 1:500 | 32KD |
| Oct4 | WB | sc-101534, Santa cruz | Mouse | 1:500 | 45KD |
| Sox2 | WB | sc-365964, Santa cruz | Mouse | 1:500 | 35KD |
| ALDH1A1 | WB | sc-374076, Santa cruz | Mouse | 1:500 | 55KD |
| c-Myc | WB | 5605, Cell Signaling Technology | Rabbit | 1:1000 | 62KD |
| Ago2 | RIP | 03-110, Merck Millipore | Mouse | 1:10 | 100KD |
| IgG | CHIP, RIP | ab18413, Abcam | Mouse | 1:10 | 150kD |
| MAML1 | WB, IP, CHIP | 11959, Cell Signaling Technology | Rabbit | 1:1000 | 120KD |
| HES1 | WB, IF | ab71559, Abcam | Rabbit | 1:1000 | 25KD |
| HEY1 | WB, IF | ab22614, Abcam | Rabbit | 1:1000 | 34KD |
| DNMT3A | WB, IF | ab232391, Abcam | Rabbit | 1:1000 | 130KD |

**Table S5. Screening of 96 predicted targets of miR-133a-3p.**

| **No.** | **Name** | **Breast cancer related** | **Migration and invasion related** | **Stemness and proliferation related** | **Reported**  **miR-133a-3p target** | **Survival related** | ***P*-value** | **Target Score（miRDB）** |
| --- | --- | --- | --- | --- | --- | --- | --- | --- |
| **1** | **MAML1** | √ | √ | √ | × | √ | 2.60E-15 | 99 |
| 2 | PTBP3 | √ | √ | √ | × | √ | 0.0053 | 74 |
| 3 | NDRG1 | √ | √ | √ | × | √ | 2.20E-09 | 55 |
| 4 | ACAT2 | √ | √ | √ | × | - |  |  |
| 5 | CAP1 | √ | √ | √ | × | - |  |  |
| 6 | FOXQ1 | √ | √ | √ | × | - |  |  |
| 7 | PTBP1 | √ | √ | √ | × | - |  |  |
| 8 | EIF4A1 | √ | √ | √ | × |  |  |  |
| 9 | FOXC1 | √ | √ | √ | × |  |  |  |
| 10 | PEAK1 | √ | √ | √ | × |  |  |  |
| 11 | PFKFB3 | √ | √ | √ | × |  |  |  |
| 12 | DUSP1 | √ | √ | × |  |  |  |  |
| 13 | EGFR | √ | √ | × |  |  |  |  |
| 14 | GABARAPL1 | √ | √ | × |  |  |  |  |
| 15 | LASP1 | √ | √ | × |  |  |  |  |
| 16 | MEIS1 | √ | √ | × |  |  |  |  |
| 17 | MSN | √ | √ | × |  |  |  |  |
| 18 | MYH9 | √ | √ | × |  |  |  |  |
| 19 | NFAT5 | √ | √ | × |  |  |  |  |
| 20 | NUP153 | √ | √ | × |  |  |  |  |
| 21 | RAPH1 | √ | √ | × |  |  |  |  |
| 22 | RBPJ | √ | √ | × |  |  |  |  |
| 23 | TAGLN2 | √ | √ | × |  |  |  |  |
| 24 | TET3 | √ | √ | × |  |  |  |  |
| 25 | TIMM17A | √ | √ | × |  |  |  |  |
| 26 | AFAP1 | √ | × |  |  |  |  |  |
| 27 | ANKRD28 | √ | × |  |  |  |  |  |
| 28 | BAZ2A | √ | × |  |  |  |  |  |
| 29 | BNIP3L | √ | × |  |  |  |  |  |
| 30 | CETN3 | √ | × |  |  |  |  |  |
| 31 | COL5A3 | √ | × |  |  |  |  |  |
| 32 | EDEM1 | √ | × |  |  |  |  |  |
| 33 | ENC1 | √ | × |  |  |  |  |  |
| 34 | FTL | √ | × |  |  |  |  |  |
| 35 | GNB4 | √ | × |  |  |  |  |  |
| 36 | HS2ST1 | √ | × |  |  |  |  |  |
| 37 | KPNA6 | √ | × |  |  |  |  |  |
| 38 | MECOM | √ | × |  |  |  |  |  |
| 39 | MEIS2 | √ | × |  |  |  |  |  |
| 40 | PIK3C2A | √ | × |  |  |  |  |  |
| 41 | PLEKHA8 | √ | × |  |  |  |  |  |
| 42 | PPP2CA | √ | × |  |  |  |  |  |
| 43 | PPP2CB | √ | × |  |  |  |  |  |
| 44 | PPP2R2D | √ | × |  |  |  |  |  |
| 45 | RARB | √ | × |  |  |  |  |  |
| 46 | RB1CC1 | √ | × |  |  |  |  |  |
| 47 | SEPHS2 | √ | × |  |  |  |  |  |
| 48 | SESN1 | √ | × |  |  |  |  |  |
| 49 | SF3B4 | √ | × |  |  |  |  |  |
| 50 | SMARCD1 | √ | × |  |  |  |  |  |
| 51 | STXBP6 | √ | × |  |  |  |  |  |
| 52 | TM9SF3 | √ | × |  |  |  |  |  |
| 53 | UBE2Q1 | √ | × |  |  |  |  |  |
| 54 | VAPB | √ | × |  |  |  |  |  |
| 55 | VAT1 | √ | × |  |  |  |  |  |
| 56 | VKORC1 | √ | × |  |  |  |  |  |
| 57 | ZC3H11A | √ | × |  |  |  |  |  |
| 58 | AFTPH | × |  |  |  |  |  |  |
| 59 | ANKRD12 | × |  |  |  |  |  |  |
| 60 | ARHGDIA | × |  |  |  |  |  |  |
| 61 | BICC1 | × |  |  |  |  |  |  |
| 62 | BTBD3 | × |  |  |  |  |  |  |
| 63 | CDK13 | × |  |  |  |  |  |  |
| 64 | CMTM6 | × |  |  |  |  |  |  |
| 65 | CNN2 | × |  |  |  |  |  |  |
| 66 | DOLPP1 | × |  |  |  |  |  |  |
| 67 | EXD2 | × |  |  |  |  |  |  |
| 68 | FAM117B | × |  |  |  |  |  |  |
| 69 | FAM160B1 | × |  |  |  |  |  |  |
| 70 | GABPB2 | × |  |  |  |  |  |  |
| 71 | GPM6A | × |  |  |  |  |  |  |
| 72 | JAZF1 | × |  |  |  |  |  |  |
| 73 | KIAA1429 | × |  |  |  |  |  |  |
| 74 | LANCL2 | × |  |  |  |  |  |  |
| 75 | MTMR4 | × |  |  |  |  |  |  |
| 76 | NUP160 | × |  |  |  |  |  |  |
| 77 | PCDHGA1 | × |  |  |  |  |  |  |
| 78 | PEX5L | × |  |  |  |  |  |  |
| 79 | PITPNM2 | × |  |  |  |  |  |  |
| 80 | RAP2C | × |  |  |  |  |  |  |
| 81 | RCE1 | × |  |  |  |  |  |  |
| 82 | RFFL | × |  |  |  |  |  |  |
| 83 | SACM1L | × |  |  |  |  |  |  |
| 84 | SEC61B | × |  |  |  |  |  |  |
| 85 | SFXN5 | × |  |  |  |  |  |  |
| 86 | SGPP1 | × |  |  |  |  |  |  |
| 87 | SGTB | × |  |  |  |  |  |  |
| 88 | SHISA5 | × |  |  |  |  |  |  |
| 89 | SLC30A7 | × |  |  |  |  |  |  |
| 90 | SLC39A1 | × |  |  |  |  |  |  |
| 91 | SNRK | × |  |  |  |  |  |  |
| 92 | TMEM167A | × |  |  |  |  |  |  |
| 93 | TMOD3 | × |  |  |  |  |  |  |
| 94 | TRAM2 | × |  |  |  |  |  |  |
| 95 | ZC3H14 | × |  |  |  |  |  |  |
| 96 | ZNF131 | × |  |  |  |  |  |  |
